# Targeting Autophagy with Natural Products as a Potential Therapeutic Approach for Cancer

**DOI:** 10.3390/ijms22189807

**Published:** 2021-09-10

**Authors:** Md. Abdul Alim Al-Bari, Yuko Ito, Samrein Ahmed, Nada Radwan, Hend S. Ahmed, Nabil Eid

**Affiliations:** 1 Department of Pharmacy, University of Rajshahi, Rajshahi 6205, Bangladesh; alimalbari347@ru.ac.bd; 2 Department of General and Gastroenterological Surgery, Osaka Medical and Pharmaceutical University, 2–7 Daigaku-machi, Takatsuki 569-8686, Osaka, Japan; yuko.ito@ompu.ac.jp; 3 Department of Biosciences and Chemistry, College of Health and Wellbeing and Life Sciences, Sheffield Hallam University, City Campus, Howard Street, Sheffield S1 1WB, UK; Samrein.Ahmed@shu.ac.uk; 4 Department of Anatomy, College of Medicine & Health Sciences, United Arab Emirates University, Al Ain 17666, United Arab Emirates; NadaA.Radwan@outlook.com; 5 Department of Hematology and Blood Transfusion, Faculty of Medical Laboratory Science, Omdurman Ahlia University, Khartoum 786, Sudan; hind.salaheldien@gmail.com

**Keywords:** autophagy, natural products, anticancer drugs, mTOR signaling, autophagy modulators, resveratrol, ω-3 PUFAs

## Abstract

Macro-autophagy (autophagy) is a highly conserved eukaryotic intracellular process of self-digestion caused by lysosomes on demand, which is upregulated as a survival strategy upon exposure to various stressors, such as metabolic insults, cytotoxic drugs, and alcohol abuse. Paradoxically, autophagy dysfunction also contributes to cancer and aging. It is well known that regulating autophagy by targeting specific regulatory molecules in its machinery can modulate multiple disease processes. Therefore, autophagy represents a significant pharmacological target for drug development and therapeutic interventions in various diseases, including cancers. According to the framework of autophagy, the suppression or induction of autophagy can exert therapeutic properties through the promotion of cell death or cell survival, which are the two main events targeted by cancer therapies. Remarkably, natural products have attracted attention in the anticancer drug discovery field, because they are biologically friendly and have potential therapeutic effects. In this review, we summarize the up-to-date knowledge regarding natural products that can modulate autophagy in various cancers. These findings will provide a new position to exploit more natural compounds as potential novel anticancer drugs and will lead to a better understanding of molecular pathways by targeting the various autophagy stages of upcoming cancer therapeutics.

## 1. Introduction

Cellular homoeostasis requires a stable balance between biosynthetic renewal and catabolic processes. Macro-autophagy, hereafter referred to as autophagy, and the ubiquitin-proteasome system (UPS) are two primarily distinct proteolytic systems in eukaryotic cells that have wide-scale degradation [1]. Given that highly selective UPS can generally only recognize short-lived protein substrates in the cell renovation system, autophagy has been well-appreciated and has followed a complex model of execution [2]. Autophagy is a self-digestive process that facilitates eukaryotic intracellular nutrient recycling via the lysosomal degradation of long-living, unwanted cellular proteins as well as damaged or defective organelles, including the mitochondria, the endoplasmic reticulum (ER), the Golgi apparatus and peroxisomes [3,4]. Autophagy has an established role in cell metabolism and energy homeostasis through the catabolism of proteins, lipids (lipophagy), carbohydrates (glycophagy) and iron (ferritinophagy), which fuels energy and nutrient stores [5]. In response to a wide variety of cellular stressors, including metabolic stress, autophagy is typically activated as a pro-survival mechanism in normal and cancerous cells [6,7]. However, in recent years, accumulated evidence has concentrated on the importance of autophagy and the variety of roles it plays in a variety of human diseases, including cancers. For instance, alterations in autophagy and inherited mutations in autophagy-related genes (ATGs) that regulate autophagy have been implicated in human cancer [4,8,9]. Autophagy has complex and context-dependent actions in cancers, and interventions to activate and suppress it have been planned as cancer therapies [4,10]. Thus, this review on natural products may reveal new therapeutic strategies that can regulate the progression of autophagy-mediated disorders, particularly cancers.

Today, although many chemotherapeutic agents have been developed to treat cancer, the effectiveness of many cancer medications remains limited or unsatisfactory. Therefore, the development of effective and non-toxic anticancer drugs or strategies is highly urgent and desirable. In the last few decades, a series of natural products with the ability to regulate physiological functions have been isolated and exploited from plants, animals and microorganisms, with most of them revealing obvious anticancer activity [11,12,13,14]. Well-tolerated and less toxic natural products will help patients to achieve better therapeutic results and will improve quality of life. Many chemotherapeutic agents have been identified by investigating potential compounds from plants, animals, and microorganisms, including marine organisms, which have been found to exert anti-cancer effects against a variety of tumors [11,12,13,14]. As a result, over 49% of approved and pre-DPA applications are natural products or agents derived from natural products, with the exception of antibodies and vaccines [15]. With respect to antitumor agents, more than 53% of anti-cancer agents applied in medicine are unaltered natural products, botanical drugs (defined mixture) and derivatives of natural products [16]. To develop an effective autophagy-targeting therapy, it is essential to identify key targets in the autophagy pathway in order to develop novel therapeutics. As discussed earlier, autophagy can play a protective or destructive role in the state of a disease, and thus it would be valuable to identify and develop pharmacologic agents that can exactly induce or inhibit this cellular process. A wide variety of potentially “druggable” targets in different stages of autophagy have been identified [4,9,17], and several natural products are capable of inducing and/or inhibiting autophagy, as discussed in this paper.

## 2. Molecular Mechanisms and Morphological Features of Autophagy

**Figure 1:**
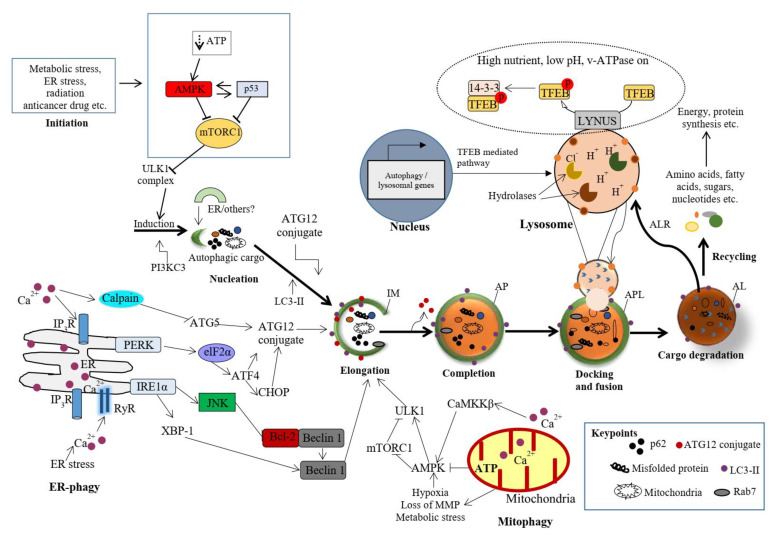
Molecular mechanisms of various stages of autophagy. Autophagy is activated in response to various cellular stresses and is triggered by a decrease in rapamycin complex 1 (mTORC1) activity due to the activation of AMP-activated protein kinase (AMPK) or p53 signaling. mTORC1 suppresses the activity of Unc-51-like autophagy activating kinase 1 (ULK1) complex. Therefore, inhibition of mTORC1 causes the initialization of the ULK1-mediated formation of the isolation (autophagosomal) membrane (IM) in association with the class III phosphatidylinositide 3-kinase (PI3K) complex (PI3KC3). The IM expands into an autophagosome (AP) with a double-layer membrane, which can engulf any cellular component, including proteins, damaged organelles and lipid droplets. The AP merges with the lysosome (via LAMP-1, 2), forming autophagolysosome (APL) or autolysosome (AL), and resulting in the degradation of the cargo by cathepsins and the autophagic lysosome reformation (ALR). The nucleation, elongation and maturation of the IM are dependent on two ubiquitin-like conjugation systems (ATG12 and ATG8), which involve multiple autophagy proteins, including Beclin1, ATG5, ATG16 and MT-associated protein 1 light chain 3 (LC3). The AL provides an acidic milieu for hydrolytic enzymes to digest the engulfed components. Nuclear localization of transcription factor EB (TFEB) is critical to the formation of lysosomes and to the enhanced expression of autophagy proteins. Importantly, autophagy could be selective of mitochondria (mitophagy) or ER (ER-phagy) [7]. However, the detailed mechanisms of this selected autophagy are beyond the scope of this study.

**Figure 2:**
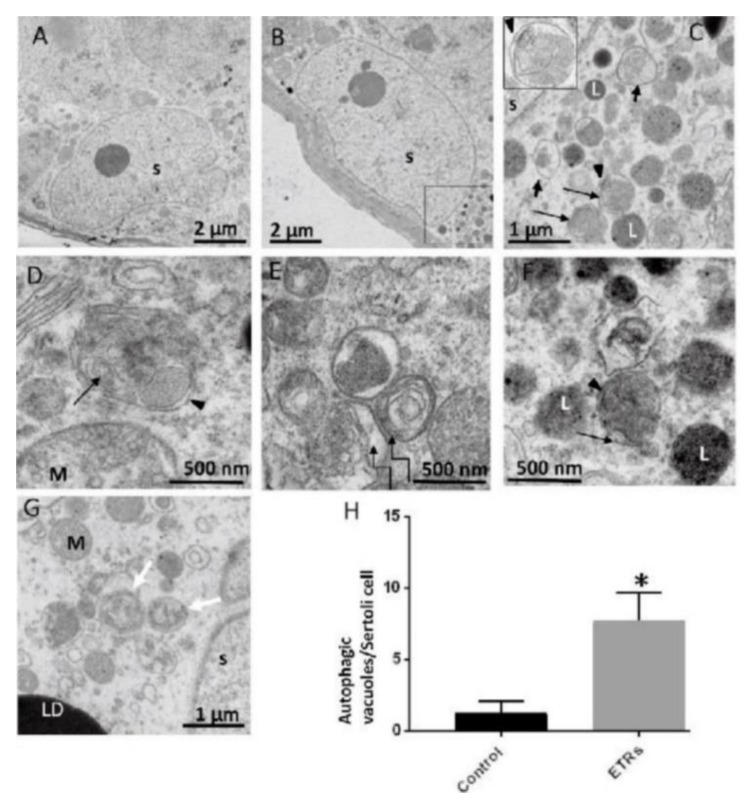
Ultrastructural features of upregulated autophagy in ethanol-treated Sertoli cells (ETR SCs). TEM of the control (**A**) and ETRs (**B**–**G**). The histogram (**H**) shows a significant increase in the number of autophagic vacuoles (AVs) in ETR SCs. The long black arrows indicate autophagosomes with a double limiting membrane (arrow heads) (magnified in the inset in (**C**)). The short arrows indicate autolysosomes. The broken arrows in (**E**) show multilamellar bodies, while the white arrows in (**G**) show autophagosomes containing fragmented mitochondria. Note the characteristic perinuclear localization of AVs. S: SC nucleus; L: lysosome; M: mitochondria; LD: lipid droplet. AVs include autophagosomes and autolysosomes. * *p* < 0.05. This was reprinted from Reference [3].

The following specific stages of autophagy are involved in the execution of the final degradation stage of recycling and energy production (Figure 1). The ultrastructural features of autophagy are shown in Figure 2. 

### 2.1. Autophagy Initiation

Autophagy is initiated by a network of complex molecular machineries that centrally involve a set of ATG proteins that modulate IM (originally termed as the phagophore) formation and autophagosome maturation. Under cellular stress, the serine/threonine protein kinase ULK1 and class III PI3K complexes are the two major protein structures involved in the initiation of autophagy in mammalian cells. It has been reported that mTORC1 inhibits the ULK1 complex, while AMPK acts as a positive regulator of ULK1 by suppressing mTORC1 in nutrient-sensing pathways (Figure 1) [18,19]. The origin of the membrane for the formation of autophagosome has been studied thoroughly, but remains an enigma in the field of autophagy [20]. While electron microscopic static images provide detailed morphological data, they do not completely explain the dynamics of autophagy, especially the sequential completion of autophagosomes and the fusion with lysosomes. Live-cell imaging techniques suggest that effective vesicular trafficking from existing compartments and membranes involves IM formation [10,21,22]. Several organelles, such as the ER [20], the Golgi apparatus, the mitochondrial outer membrane (MOM), endosomes, the plasma membrane and the nuclear envelope [23], may donate membranes for IM formation. It was also found that the specialized ER regions called ER exit sites (ERES) are important mediators for IM formation [20]. Moreover, several inter-organelle-contact sites have emerged as dynamic spots for the lipid transition between the membranes and the growth of nascent IM. ER–mitochondria contact sites, also known as mitochondria-associated ER membranes (MAMs), are claimed to be the source of IM [24,25]. MAMs spatially overlap with specialized membrane compartments, called omegasomes (omega-shaped structures), and these serve as a cradle of IM formation and autophagosomal vesicles close to the ER.

### 2.2. Cargo Nucleation, Elongation and Enclosure of IM

During the initiation of autophagy, the localization of the ULK1 complex in the IM site regulates the vesicle nucleation machinery. The nucleation machinery of the initial IM is dependent on local phosphatidylinositol 3-phosphate (PI3P) production, which is marked by its binding protein, i.e., the double FYVE domain-containing protein 1 (DFCP1) by the class III PI3K complex. Thus, vesicle nucleation involves the ULK1 complex mechanistic link core complex, which consists of class III PI3K (PIK3C3), Beclin1, p150, ATG14L and the activating molecule in Beclin1-regulated autophagy protein 1 (Ambra1) [26,27]. The elongation step of the IM is controlled by two evolutionarily conserved ubiquitin-like (UBL) conjugation systems called the ATG12–ATG5–ATG16 complex (also known as the ATG12 conjugation system) and the LC3-phosphatidylethanolamine (LC3-PE) conjugation system [28,29] (Figure 2).

**Figure 3:**
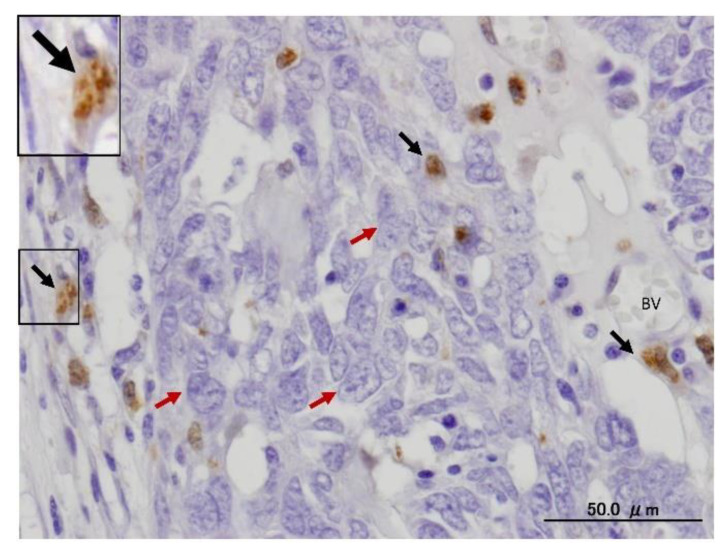
Expression of LC3 in stromal cells of serious human ovarian carcinoma using IHC. The black arrows indicate LC3-II puncta in stromal cells, whereas the red arrows mark ovarian cancer cells. The framed area is magnified in the inset. BV, blood vessel. The avidin biotin complex (ABC) IHC method is performed. The diaminobenzidine (DAB) is used as a chromogen (brown reaction), whereas the hematoxylin (blue) is applied for nuclear counterstaining.

In response to various stressors, such as starvation, oxidative stress and hypoxia, specific cargos are sequestrated with autophagy receptors in IM. The autophagy receptors bind specifically to ubiquitinated cargos that are tagged with degradation signals of the autophagy machinery through their LC3 interacting regions (LIR) [30,31]. In general, the LIR motifs interact with autophagy regulatory proteins of the LC3/GABARAP family [32]. In mammalian cells, more than twenty autophagy receptors have been identified, and sequestosome-1 (p62) is one of the most common autophagy receptors [33]. LC3-II is the major autophagy marker, and its upregulation is an indicator of autophagy activation and the formation of dual-membrane autophagosomes. Using immunofluorescence and immunohistochemistry (IHC) methods, the autophagosomes appear as dots or puncta, indicating the expression of LC3-II, which could be detected as shown in Figure 3 [3,7]. An autophagic flux involves the formation of autophagosomes, their administration to lysosomes, and the subsequent degradation and release of degraded macromolecules into cytosol, which are then recycled. This is called productive or completed autophagy. Thus, the increases in the level of LC3-II, as evidenced by the accumulation of autophagosomes, are not measurements of the autophagic flux per se, but may reflect the induction of autophagy sequestration and/or the inhibition of autophagosome clearance, which results from the fusion failure with lysosomes or lysosomal dysfunction. This incomplete or impaired autophagic flux may result in cell death, such as apoptosis or cell death with autophagic features (sometimes called autophagic cell death). Autophagic flux can be monitored through the use of inhibitors such as choloroquine (CQ), bafilomycin A1 (Baf A1) or lysosomal protease inhibitors. This can be determined by measuring the levels of LC3-II in both the presence and absence of saturating levels of inhibitors; if flux is occurring, the amount of LC3-II will be higher in the presence of the inhibitor [3,7,8,9,10].

### 2.3. Transport of Autophagosomes

Autophagosomes (double membrane vesicles) are randomly formed throughout the cytosol, concentrating and continuously moving during the maturation process. In general, microtubules (MTs) serve as an interconnected network of intracellular movement powered by specific motor proteins, including the kinesin and dynein protein families [34]. Kinesins normally transport the cargoes to the peripheral plus-ends of MTs, whereas dyneins, a class of AAA+-ATPase-associated motors, are involved in the delivery of autophagosomes towards the minus-end [35]. The coordinated transport of lysosomes and autophagosomes in the perinuclear zone is necessary for the adequate fusion of these two organelles [36,37], as shown in Figure 2. Under various stress conditions, the intracellular pH increases, resulting in the relocation of lysosomes to the perinuclear region, where mature autophagosomes are transported to the same region by the MTs [3,36]. Consequently, the lysosomes in the perinuclear areas merge with mature autophagosomes in order to acidify them [3,38]. This is shown in Figure 2.

### 2.4. Autolysosome Formation, Vesicle Degradation and ALR Cycle

**Figure 4:**
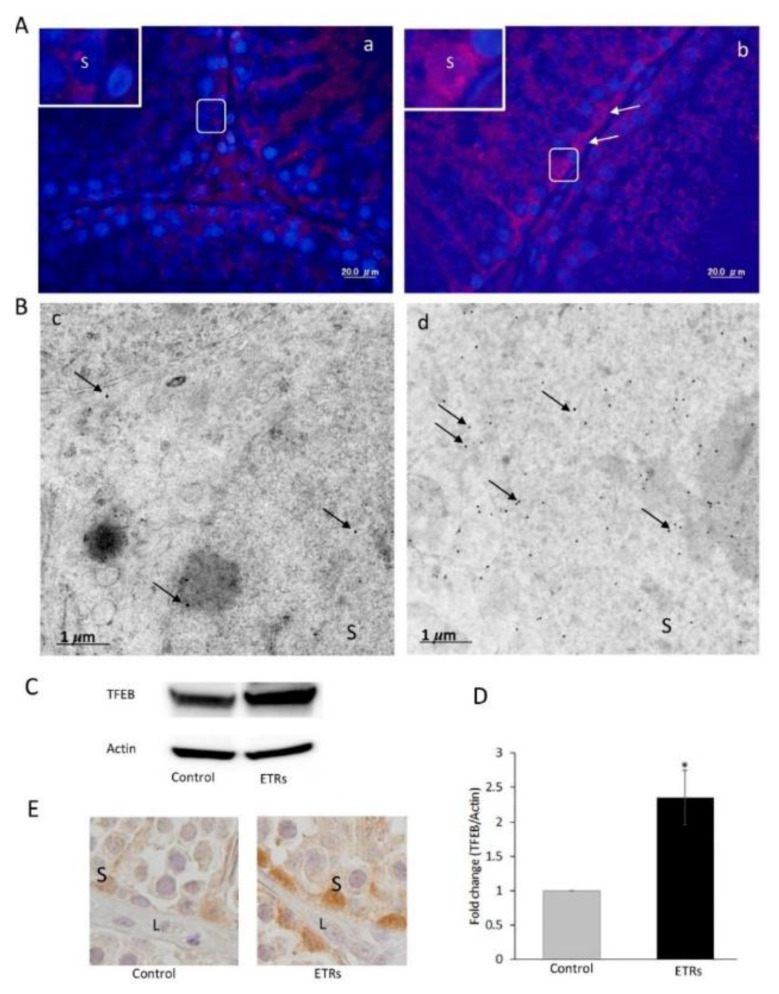
Elevated expression and nuclear translocation of TFEB in ETR SCs. (**A**) The IF of TFEB expressions in the control (a) and ETRs (b). The insets present higher magnifications of the framed areas. Note the overexpression of TFEB (white arrows) in the SC nuclei of ETRs. (**B**) Immunogold labeling of TFEB (black arrows, 15 nm gold particles) in the control (c) and ETR SCs (d). (**C**) Western blot of TFEB in the control and ETR tests (n = 3). (**D**) Histogram showing a significant increase in TFEB expression in the ETR tests. * *p* < 0.05 (*t*-test). (**E**) IHC showing TFEB nuclear translocation in ETR SCs (part of a seminiferous tubule), confirming the IF and IEM results (**A**,**B**). S: SC nucleus; L: Leydig cell. This was reprinted from Reference [43].

As the mature autophagosome docks to the lysosome, a single-membrane autolysosome is formed [39]. Merging autophagosomes with late endosomes to form amphisomes, prior to fusion with the lysosome, is also reported to increase cargo delivery and reduce the pH of autolysosomes [40]. Upon fusion with mature lysosomes, the intralysosomal contents degrade with the release of end products into the cytosol, producing local nutrient availability as a source of cellular energy. This process results in the reactivation of mTOR, a key regulator of autophagy, and the regeneration of mature lysosomes from autolysosomes, which is a process called ALR [41,42]. While there is significant insight into each of these stages of autophagy, the molecular mechanisms controlling the biogenesis of autophagosomes, autolysosomes and lysosomes are complex. In response to intracellular and environmental stressors, autophagy is primarily regulated by two critical signaling pathways, i.e., the mTOR-dependent and mTOR-independent signaling pathways, which include Ca^2+^, inositol 1,4,5-trisphosphate (IP3) receptor (IP3R), AMPK, stress activated enzyme c-Jun N-terminal kinase (JNK), and B-cell lymphoma 2 (Bcl-2) homology (BH) domain 3 (BH3)-only proteins [42]. It has been reported that nuclear translocation of TFEB positively regulates the formation of lysosomes and enhances the expression of autophagy proteins, specifically, under starvation, oxidative and nitrative stress (Figure 4). Using various methods, we recently provided evidence of the enhanced nuclear translocation of TFEB in the SCs of ETRs, which is correlated with the upregulation of autophagy and mitophagy proteins [43], as shown in Figure 4.

## 3. Natural Products as Inhibitors of Autophagy in Cancer

Autophagy is mandatory to maintain cell homeostasis. In healthy cells, this homeostatic activity provides a strong barrier against oncogenesis. As a result, many oncoproteins inhibit and several oncosuppressor proteins promote autophagy. In addition, autophagy might contribute to oncogene-induced cell death or oncogene-induced senescence, which are two fundamental oncosuppressive mechanisms. Additionally, autophagy is necessary for optimal anticancer immunosurveillance. However, autophagy enhances the progression of established cancers via multiple mechanisms, and pharmacological inhibitors of autophagy may have robust antineoplastic effects, at least in certain contexts. Enhanced autophagy in the stromal compartment of pancreatic cancers supports tumor growth via autophagy-mediated secretion of the nonessential amino acid alanine, thus fueling the mitochondrial metabolism of cancer cells and allowing them to thrive in an austere microenvironment [8,9,10]. Therefore, as shown in Figure 3, the enhanced expression of LC3-II in the stromal cells of ovarian cancer may fuel the growth of cancer cells. Natural products have historically been regarded as different doctrines of traditional medicine or folk medicine for the management and treatment of a variety of human diseases, including cancer, by inhibiting autophagy pathways [4,44,45]. Several studies have shown that the inhibition of autophagy using natural compounds can effectively enhance the cancer cell death induced by diverse anticancer drugs. Importantly, the use of natural products in the suppression of autophagy can be carried out by the specific targeting of various structures in the autophagy pathway (isolation membranes, autophagosomes, autolysosomes and lysosomes), as shown in Figure 1.

### 3.1. Class III-PI3K Complex Inhibitors

Class III PI3K mediates the production of PI3P, a key lipid-signaling molecule that is known to be required for autophagosome formation via the recruitment of autophagy machinery at the IM (Figure 1). Evidence has confirmed that class III-PI3K inhibitors interfere with the formation of autophagosomes. The main inhibitors for the early stages of autophagy, including the mechanism of action (M/A), are given in Table 1.

Based on their blocking effect on class III PI3K activity, the natural product wortmannin [46,47] and its concurrent synthetic compounds, 3-methyladenine (3-MA), KU55933 and LY294002, are well recognized as early stage autophagy inhibitors in the literature [190]. Wortmannin was originally identified as a potent inhibitor of the neutrophil respiratory burst and smooth muscle myosin light chain kinase (MLCK) [48]. Later on, it has become clear that wortmannin is a more potent inhibitor of the PI3K superfamily than of the MLCK. More recently, wortmannin has also been reported to suppress polo-like kinase 1 (PLK1) [191], mTOR [49], DNA-dependent protein kinase (DNA-PK) [192] and ataxia–telangiectasia mutated (ATM) [193] at micromolar concentrations [194]. Unlike 3-MA, wortmannin is a more practical and feasible autophagy inhibitor due to more persistent suppression on class III PI3K, regardless of the nutritional status. While 3-MA, wortmannin, and LY294002 have been useful in many contexts to inhibit autophagy and to eventually sensitize tumor cells to death, they also target class I PI3Ks. As non-specific inhibitors of PI3K, these compounds affect several cell processes and are toxic after long-term exposure [195]. Moreover, these compounds have been shown to play a role in many other cellular processes, including fluid-phase endocytosis and cell migration [196]. Thus, these compounds have been unsuitable for clinical applications due to their inherent toxicity, poor solubility and low stability [33]. However, nanoparticle (NP)-based drug delivery of wortmannin reduces its toxicity and increases its solubility [197], and thus NP-wortmannin acted as a potent radiosensitizer in vitro and in vivo in a mouse xenograft model of cancer. This indicates that the administration of wortmannin nanoparticle medications may have clinical applications [197]. Interestingly, a synthetic derivative of wortmannin, PX-866 (sonolisib), is a potent pan-isoform inhibitor of PI3K that blocks temozolomide-induced autophagy and promotes apoptosis in glioblastoma cells [50]. Treatment with PX-866, in combination with docetaxel, is well tolerated without evidence of cumulative toxicity and controls disease progression in patients with advanced solid tumors. Furthermore, PX-866 was identified as completing several clinical trial evaluations (Identifier: NCT01331083, NCT01204099, NCT01252628, and NCT01259869) for the treatment of patients with recurrent or metastatic cancer [51,52,53]. Several reports have shown that cycloheximide is a fast and effective inhibitor of the early stages of autophagy [54,55]. However, cycloheximide is generally used only for autophagy inhibition in vitro because it is not suitable for human use due to significant toxic side effects, including DNA damage and teratogenesis [55].

Petrosaspongiolide M (PSM) exerts inhibitory effects on autophagy in the human histiocytic lymphoma (U937) cells by downregulating Beclin1 levels. As an immunoproteasomal inhibitor, PSM binds the active sites in the inner core of proteasome in U937 cells and accumulates ubiquitinated proteins, as well as p53, which is a regulator of the cell-cycle and cell death [56]. In this regard, PSM represents an interesting molecule for the modulation of intracellular proteolysis through the dual inhibition of proteasome and autophagy [57]. Epirubicin (EPI) is an anthracycline drug that has been widely used to treat bladder cancer. As a topoisomerase-II inhibitor, EPI causes apoptosis in cancer cells by inducing DNA damages. However, resistance to EPI becomes a great challenge in treating bladder cancer because it induces cytoprotective autophagy in bladder cancer cell lines T24 and BIU87 via the activation of JNK-mediated phosphorylation of Bcl-2 and disruption of the Bcl-2/Beclin1 complex [198]. The green tea derivative tea polyphenol (TP) displays strong biological effects, including anticancer properties. TP inhibits EPI-induced autophagy and promotes EPI-induced apoptosis in human bladder cancer cells [198]. Harmine analogues have long been considered to be anticancer agents due to their reported anti-proliferative activity. While harmines seem to cause DNA damage and inhibits cellular enzymes, the precise mechanism of action of harmines remains elusive. It has been found that N2-benzyl and N9-arylated alkyl, which are analogues of harmine, strongly inhibit the growth of cancer cells that originate from the breast, lung, bone and pancreas, but not that of normal fibroblasts via the induction of apoptosis and inhibition of autophagy by reducing the conversion efficiency of LC3-I to LC3-II [58].

### 3.2. IM Elongation and Enclosure Stage Inhibitors

Since MTs have a major role in autophagy pathways in non-mitotic cells, MTs may be effective targets in cancer cell death. The MT targeting agents (MTAs) of natural drugs have shown potential therapeutic benefits in cancers [59]. Many natural agents and/or their MTA analogues may bind to the tubulin and alter the assembly properties used in tumors by inhibiting mitosis. Based on the role of the MT network in autophagy, pharmacological MTAs are classified into two main groups. The first group is microtubule-destabilizing agents (or antipolymerization drugs), such as the vinca alkaloids (vinblastine, vincristine, vinorelbine, vindesine and vinflunine), colchicine, cryptophycins, halichondrins, estramustine and combretastatins, which are used clinically or are under clinical investigation for the treatment of cancer [59]. The second group is MT-stabilizing agents, which stimulate MT polymerization, with examples including paclitaxel (Taxol), docetaxel (Taxotere), the epothilones, and discodermolide.

Vinca alkaloids are considered “wonder drugs” for fighting cancer [13,60]. For example, they are used in the treatment of childhood hematologic malignancies (leukemia). Vinca alkaloids vinblastine or vincristine are able to depolymerize the whole MT network (both acetylated and non-acetylated forms) and reduce the conversion efficiency of LC3-I to LC3-II. These alkaloids contribute to their anticancer activity by preventing autophagosome formation and maturation [60]. At higher concentrations, vinblastin reduces the autophagic marker p62 and completely inhibits the merging of autophagosomes with lysosomes [60].

Colchicine was approved by the Food and Drug Administration (FDA) in 2009 for the treatment of gout attacks and familial Mediterranean fever. Previous studies have shown that colchicine induces autophagy and senescence in lung cancer cells at a clinically acceptable level. However, extensive research studies suggest that it prevents autophagosome formation by inhibiting MT polymerization as a tubulin binder and thus acts as mitotic spindle poison in cells. Combretastatins exhibit cytotoxic properties and inhibit tubulin polymerization in cancer cells in vitro [60]. Combretastatin A-4 (CA-4, also known as fosbretabulin), the most potent member of this family, has a great effect in antitumor therapy and has entered several clinical trials for solid tumors. CA-4 phosphate (CA-4P), a water-soluble prodrug of CA-4, has also progressed into clinical trials for the treatment of solid tumors (Table 2). Since CA-4 has a high affinity to tubulin and destabilizes the tubulin polymers of the cytoskeleton, it is also used as an angiogenesis inhibitor and a vascular disrupting agent [60,61]. Some reports have shown that CA-4 induces prosurvival autophagy in both human osteosarcoma [62] and adenocarcinoma cells [63]. However, evidence has indicated that the suppression of autophagy has been suggested to potentially enhance the therapeutic efficacy of CA-4 [63,64].

As an antioxidant agent, N-acetyl cysteine (NAC) effectively abolishes oxidative stress markers, such as intracellular reactive oxygen species (ROS) [65], and alters the cellular redox status. NAC therefore plays an important role in triggering apoptosis and inhibiting the autophagy induced by starvation, trehalose and recombinant human arginase (rhArg) in COS-7 and HeLa cells. NAC also induces apoptosis in colon carcinoma cells by increasing the pro-apoptotic Bax levels and by increasing susceptibility to the chemotherapeutic agent 5-fluorouracil (5-FU) [66].

The natural valosin inhibitor containing protein (VCP or p97) xanthohumol (XN) has been considered for its potentially beneficial effects in HeLa cells, including the inhibition of diacylglycerol acyltransferase, induction of apoptosis, as well as the inhibition of autophagy via the upregulation of p62 and LC3-II [67].

To date, over 10 different salvianolic acids have been identified and referred to: salvianolic acid A, B, C, D, E, F, G, etc. Salvianolic acid A (Sal A) and salvianolic acid B (Sal B) are the most abundant compounds among salvianolic acids. Both in vitro and in vivo, most of the salvianolic acids showed anti-inflammatory and antioxidative effects [68]. Some studies predict that Sal A and Sal B will have therapeutic effects on breast cancer, lung cancer and liver cancer. Sal A reverses the resistance of circulating cancer cells (CCC) in breast cancer MCF-7/PTX cells to paclitaxel [69,70]. Sal A potentially reduces A549 lung cancer cell growth, and promotes apoptosis by enhancing the expression of phosphatase and the tensin homolog deleted on chromosome 10 (PTEN) and localized on the cytoplasmic membrane, which in turn inhibits PI3K signaling. Sal A inhibits the growth of mouse lung cancer cells by inhibiting the expression of c-myc and JNK [68]. Sal B, combined with other compounds, inhibits migration, invasion and the epithelial–mesenchymal transition (EMT) process of A549 cells by PTEN/PI3K/protein kinase B (PKB or AKT) [71]. Additionally, Sal B inhibits the proliferation of breast cancer cells and promotes their apoptosis [68]. Sal B reduces the incidence of squamous cell carcinoma (SCC) by inhibiting angiogenesis and decreasing the expression of hypoxia-inducible factor 1α (HIF-1α) and vascular endothelial growth factor (VEGF) [68]. Sal B exerts some inhibitive effects on lung cancer [72] in vivo. Sal B could activate apoptosis in human hepatocellular carcinoma (HCC) through the mitochondrial pathway [73]. Interestingly, Sal B induces autophagy in both hepatoma cells and colorectal cancer (CRC) cell lines. The Sal B-induced autophagy, which is mediated by the AKT/mTOR signaling pathway, can play a pro-apoptotic role in cancer cells [73]. It is also reported that Sal B inhibits the early stages of autophagy and interferes with the development of autophagosome via the inhibition of LC3 lipidation and by blocking the elongation of IM [68,73].

Deguelin effectively inhibits autophagy in several types of cancer, including pancreatic cancer, by blocking LC3 lipidation. Deguelin induces incomplete autophagy in pancreatic cancer cells by inhibiting autophagy flux, as evidenced by the impairment of autophagosome maturation and the subsequent accumulation of LC3-II and p62 in dose- and time-dependent manners [74]. Doxorubicin-induced autophagy (Dox) plays a pro-survival role in pancreatic cancer cells; thus, the pharmacological inhibition of autophagy by QC or the silence of ATG5 enhances Dox-induced cancer cell death. Similarly, deguelin’s inhibition of autophagy has also chemosensitized pancreatic cancer cell lines to Dox [74]. However, a previous study showed that deguelin may induce both apoptosis and autophagy in cultured head and neck SCCs. This is mediated by the inhibition of AKT signaling, the downregulation of survivin and cycline-dependent kinase 4 (Cdk4) expression, as well as the disruption of their association with heat shock protein 90 (Hsp-90) [75].

### 3.3. Docking and Fusion Stage Inhibitors

Polyether ionophores, such as monensin and nigericin, are produced by Streptomyces species and are potent antimicrobial and anticancer agents that belong to a large class of naturally occurring polyketides [76]. A number of studies have revealed that monensin and nigericin have been linked to autophagy and cell death via the interference from the fusion of autophagosome and lysosome, and thus blocks the maturation of the autophagic process. These two compounds block autophagic flux, resulting in the accumulation of autophagy flux markers LC3-II and p62, along with the cleavage of caspase-3, caspase-9 and poly(ADP-ribose) polymerase 1 (PARP-1), which is a hallmark of caspase-dependent apoptosis [77,78]. In addition, another polyether antibiotic, salinomycin, is reported to inhibit autophagic flux in several cancer cell lines [199].

Asparagine-rich foods include dairy products, whey, beef, poultry, eggs, fish, seafood, asparagus, potatoes, legumes, nuts, seeds, soy, and whole grains. Low asparagine-containing foods mostly include fruits and vegetables, which can slow down breast cancer metastasis [79]. Mice harboring primary 4T1 mammary tumors treated with L-asparaginase or fed with a low-asparagine diet experienced a decrease in metastases with no effect on primary tumor growth. This can be attributed to blockage of the fusion of autophagosomes with lysosomes and the suppression of lysosomal functions [79]. Additionally, L-asparaginase has been reported to be an important drug for the treatment of acute lymphoblastic leukemia (ALL) cells in the last few decades [80]. Contradictory reports indicate that L-asparaginase catalyzes the conversion of L-asparagine to aspartic acid and ammonia, resulting in the deprivation of circulating asparagine. This leads to metabolic stress, as evidenced by the inhibition of both glycolysis and oxidative phosphorylation, and the activation of autophagy in ALL cells [81].

Liensinine suppresses autophagic degradation by blocking autophagosome–lysosome fusion and the subsequent accumulation of autophagosomes in breast cancer cells. While the inhibitory effect on autophagy is similar to CQ and Baf A1, liensinine’s blockage of autophagosome–lysosome fusion differs from these inhibitors. CQ and Baf A1 cause alkalinized lysosomal pH, suppress the fusion process, and impair the action of lysosomal hydrolases, whereas lysosomal pH is unaffected in response to liensinine treatment. Thus, lysosomal pH may not be necessary to inhibit autophagosome–lysosome fusion by liensinine [200]. Interestingly, liensinine interferes with the recruitment of the small binding protein GTP RAB7A in lysosomes, but not in autophagosomes, and suppresses the transport of endocytic cathepsins to lysosomes and finally stops autophagosome–lysosome fusion. Additionally, the combination of liensinine and Dox causes the synergistic inhibition of the viability and induction of death in breast cancer cells due to altering autophagy/mitophagy by liensinine [200]. A recent and contradictory study found that the bisbenzylisoquinoline alkaloids neferine, liensinine and isoliensinine inhibit cell growth and exhibit significant anti-migration activities in prostate cancer cells. They induce apoptosis and autophagy by activating cleaved caspase-9, cleaved PARP, Bax, and LC3-II, but reduced the expression of Bcl-2 and PARP proteins in LNCaP cells in short-term treatments (24 h) [201].

Oblongifoline C (OC) and guttiferone K (GUTK) are the major active components of the *Garcinia yunnanensis* Hu fruit with anticancer activities, but they act through various mechanisms [82]. OC promotes apoptosis and inhibits autophagy and cancer metastasis [83,84]. Similarly, GUTK activates apoptosis, arrests the cell-cycle, and promotes autophagy [85,86]. However, OC and GUTK show synergistic inhibition on colorectal cancer (CRC) HCT116 cells. Moreover, the combination of OC and GUTK markedly increases the cleavage of casapse-3, enhances cellular ROS production and upregulates JNK protein phosphorylation, resulting in autophagy initiation. OC acts as a powerful autophagic flux inhibitor by blocking autophagosome–lysosome fusion and increases the pH in acid compartments. In addition, OC inhibits lysosomal proteolytic activity and downregulates lysosomal cathepsins. Importantly, OC efficiently sensitizes nutrient-deprived cancer cells to caspase 3-dependent apoptosis in vitro [82].

### 3.4. Late-Stage Disruptors

Currently, most of the natural compounds are currently used to inhibit autophagy at a late phase of autophagy. For instance, lysosomotropic agents (QC), vacuolar-type ATPase inhibitors (V-ATPase) (Baf A1) and lysosomal-type protease inhibitors (pepstatin A) all interfere with the final steps of the autophagy pathway. These compounds inhibit the degradation of autolysosome by lysosomal enzymes, leading to cytoplasmic accumulation, which may be toxic to cells. Since lysosomes are involved in many biological processes besides autophagy (e.g., endocytosis), these molecules have multiple off-target effects.

#### 3.4.1. Acidification Stage Inhibitors

The quinine extracted from the Cinchona bark, and its synthetic analogue CQ (orginallyt used as antimalarial agents), are repurposed for cancer treatment. Thus, these drugs, including the antimalarial quinine, CQ, and its derivatives, block the acidification processes of autolysosome and lysosomes in cancer cells, resulting in a reduction in autophagic flux and cell death [202].

As autophagy plays an essential role in effective cellular response in host defense against mycobacterial NTM infections, the use of autophagy blockers, such as azithromycin in patients with chronic inflammatory lung diseases, may result in highly pathogenic and fatal infections [87]. Therapeutic doses of azithromycin suppress the clearance of autophagosomes by altering lysosomal acidification and autophagic degradation in macrophages, resulting in a failure to kill intracellular mycobacteria and the persistence of lung infections [87]. Clarithromycin strongly attenuates the late stages of the autophagy process in myeloma cells by halting the fusion of autophagosomes with lysosomes and altering lysosomal acidification, causing the induction of cell death [88].

Matrin shows inhibitory effects on proliferation and metastasis, and causes apoptosis in a variety of malignant cells, e.g., C6 glioma cells. As an autophagy inhibitor, matrine elevates pH values in endosomes/lysosomes, which in turn inhibits trafficking and lysosomal proteases in human gastric cancer cells [89]. Recent contradictory evidence has shown that matrine induces autophagy in human hepatoma cells with inactive p53 by the induction of the AMPK signaling pathway [90]. Martine-derived MASM, a potent derivative of matrine, possesses potency against cancer cells by inducing autophagy and apoptosis through ROS-mediated PI3K/AKT/mTOR and the extracellular signal-regulated kinase1/2 (ERK1/2)/p38 signaling pathway in epithelial cancer cell lines [91]. Thus, the action of matrine is cell-specific and signaling pathway-dependent.

Elaiophylin acts as an autophagy inhibitor because it disrupts lysosomal degradation. It blocks autophagic flux in the late stages of autophagy in ovarian carcinoma cells. Elaiophylin usage could promote a substantial accumulation of autophagosomes [92]. Additionally, elaiophylin abrogates the maturation of cathepsins B and D and induces subsequent lysosomal membrane permeabilization (LMP). Elaiophylin decreases cell viability and induces cell death via the inhibition of autophagy and sensitizes the antitumor effect to cisplatin in vitro. Administration of elaiophylin (dose 2 mg/kg) displays a significant antitumor effect without toxicity [92]. Another report suggests that elaiophylin exerts anti-myeloma activity by blocking autophagy flux, inducing apoptosis and arresting proliferation in multiple myeloma cells [93].

An anti-schistosome agent, lucanthone, impairs autophagy by inducing the accumulation of p62 and disrupting lysosomal functioning. In addition, lucanthone stimulates apoptosis via cathepsin D accumulation and potentiates the histone deacetylase inhibitor and vorinostat-mediated cell death [94]. Lucanthone, in association with chemotherapeutic agents, has already reached clinical trials (Table 2) and is currently in phase II clinical trials for glioblastoma multiforme.

#### 3.4.2. Vacuolar-Type H^+^-ATPase (V-ATPase) Inhibitors

V-ATPase is found in the membranes of many organelles, including lysosomes, endosomes and secretory vesicles, where they play a variety of vital roles in many cellular processes, and its dysregulation leads to the maintenance of the acidic milieu, thus causing several diseases, such as osteoporosis and cancer [203].

Macrolide antibiotics bafilomycins A1, B1, D, F, G, H, I and J are potent autophagy inhibitors that function via the induction of autophagosomes accumulation. Baf A1 is considered to be a selective prototypical V-ATPase inhibitor at low nanomolar concentrations and is therefore often used (Baf A1 clamp assay) to block late autophagic flux [95]. Baf A1 may also target early stages of autophagy by activating mTOR signaling, thus disassociating the Beclin1-class III complex and inhibiting autolysosomal formation [96]. Thus, Baf A1 prevents activation of lysosomal enzymes via blocking its acidification process [97]. In addition, Baf A1 induces the binding of Beclin1 to Bcl-2, which further inhibits autophagy and promotes apoptotic cell death [96]. Baf A1 also targets mitochondria and induces caspase-independent apoptosis by inducing the translocation of apoptosis-inducing factors from mitochondria to the nucleus [96]. Another selective V-ATPase inhibitor, concanamycin-A, also increases the accumulation of autophagosomes [98]. Concanamycin A shows significant global toxicity due to the inhibition of the V-ATPase in several tissues. It has been reported that manzamine A has shown anticancer activity against pancreatic cancer cells through the inhibition of V-ATPase and autophagy through the accumulation of lysosomes/autolysosomes [99]. For example, manzamine A is active against AsPC-1 pancreatic adenocarcinoma cells by reducing cell dissociation, abrogating cell migration, and sensitizing cells to apoptosis [100].

Dox delivers antitumoral activity in two basic ways: through interference with DNA synthesis and through the induction of its damage [101]. Dox also blocks autophagic flux by impairing lysosome acidification through the suppression of V-ATPase activity and lysosomal function. Despite its highly beneficial effects against cancer, its clinical uses are limited by its severe side effects, such as life-threatening cardiotoxicity, particularly in children with cancer. Since intracellular Ca^2+^ signaling has been reported to play an important role in the regulation of autophagy, Dox causes the significant abnormal accumulation of cytosolic Ca^2+^ in human cardiac progenitor/stem cells and causes cardiotoxicity [102]. Another natural V-ATPase inhibitor, cleistanthin A, has shown cytotoxicity in several tumor cell lines. Archazolid, another well-investigated V-ATPase inhibitor, reduces protease activity, such as B-cathepsin in vitro and in vivo [103].

#### 3.4.3. Lysosomal Hydrolytic Enzyme Inhibitors

Leupeptin impairs amphisome–lysosome fusion and suppresses cathepsin B, H and L [104]. Leupeptin also inhibits reversible trypsin-like serine proteases and most cysteine proteases (including trypsin, papain, cathepsin B and calpain) [103]. Pepstatin A is an inhibitor of acid proteases (aspartyl peptidases). It forms a 1:1 complex with proteases such as pepsin, renin, cathepsin D, bovine chymosin, and protease B [105].

## 4. Natural Products as Inducers of Autophagy in Cancer

In the following section, the main natural products are found to activate autophagy (Table 1) and consequently modulate cancer cells in various models. The molecular targets (where known) for these mammalian cells are shown in Figure 1.

### 4.1. Initiation Stage Activators: mTOR Inhibitors

Caloric restriction (CR) or fasting is one natural and effective phenomenon that induces autophagy, as it activates multiple regulatory pathways. For example, CR results in the inhibition of mTORC1 and the activation of AMPK, which in turn activates the autophagy-promoting ULK1 complex (Figure 1) [106]. Furthermore, CR stimulates sirtuin 1 (SIRT1), which deacetylates and thereby activates essential autophagic proteins [106].

While the inhibitor mTOR rapamycin (sirolimus) is known to be a powerful autophagy inducer that extends the lifespan of various organisms, from flies to mammals [106,107,108], it has serious adverse effects, including insulin resistance [106]. Most of these side effects have been attributed to the chronic inhibition of mTORC2 [204]. Consequently, considerable effort has been devoted to discovering specific mTORC1 inhibitors, such as semi-synthetic analogues of rapamycin (known as rapalogs), including temsirolimus, everolimus, deforolimus, zotarolimus, biolimus, WYE-592 and ILS-920 [205], which have fewer side effects. These rapalogs are allosteric selective inhibitors of mTORC1 that affect downstream targets, including the activation of autophagy [206]. However, their efficacy in inhibiting tumor growth is limited due to the lack of inhibition of mTORC2 and other compensatory signaling pathways that promote cell survival [206]. Mechanistically, rapamycin is an allosteric inhibitor of mTOR and only suppresses part of the mTORC1 function, whereas both PP242 and Torin1 are catalytic inhibitors that are able to completely suppress both mTORC1 and mTORC2 via binding to ATP-binding sites [206].

### 4.2. Polyphenolic Compounds

Polyphenolic compounds are the most common bioactive secondary plant metabolites that are present in fruits, vegetables, seeds, and others, and they have a wide range of activities in the prevention and treatment of various diseases, including cancers [207]. Several of the beneficial effects of polyphenols have been attributed to their antioxidant activity [208]. Additionally, polyphenols affect numerous cellular targets that can modulate autophagy, which interferes with the symptoms and putative causes of cancers [208]. Today, it is widely accepted that dietary flavonoids, including the commonly occurring flavonols quercetin and kaempferol, the flavones apigenin and luteolin, green tea catechins, and the isoflavone genistein, have strong anticancer potentials, thus exerting antiproliferative, cytotoxic, proapoptotic, anti-inflammatory, antiangiogenic, antimetastatic, and antiinvasive activities. The strong potential of these compounds in the fight against cancer has been proven in numerous experimental studies, in both in vitro cell cultures as well as in animal (rodent) models [208,209,210].

#### 4.2.1. Quercetin

Quercetin is a well-known antioxidant flavonoid that has antitumor effects [109]. Quercetin induces autophagy in different cancer cells through the modulation of the AKT-mTOR signal pathway [110]. Quercetin triggers autophagy by AMPK activation and the accumulation of HIF-1α, which represses mTOR signaling and induces the expression of Bcl-2/adenovirus E1B 19 kDa protein-interacting protein 3/ligand (BNIP3/BNIP3L) to disrupt the Beclin1/Bcl-2 (Bcl-xL) complex [110]. Poly(DL-lactide-co-glycolide) quercetin nanoparticles stimulate autophagy and cell death through the suppression of the AKT/mTOR signaling pathway in human neuroglioma cells [110]. Quercetin induces ER stress, activates protective autophagy and apoptosis, and simultaneously stimulates signal transduction and the activation of the transcription axis 3 (p-STAT3)/Bcl-2 in ovarian cancer. As a chemopreventive agent, quercetin plays an important role in modulating chemotherapeutic drug sensitivity [110]. Quercetin also regulates apoptosis and autophagy-related pathways and facilitates gemcitabine (an analog of deoxycytidine for DNA synthesis inhibition) chemosensitivity through the receptor involved in advanced glycation end products (RAGE)/PI3K/AKT/mTOR axis in human pancreatic cancer cells. Quercetin also suppresses multidrug resistance protein 1 (MDR1) expression, blocks drug efflux via P-glycoprotein (P-gp) transport proteins, and increases the activity of anti-cancer drugs in uterine sarcoma MES-SA cells [110]. Quercetin-induced initial autophagy in gastric cancer protects cancer cells from late apoptosis [211]. Rutin, also called rutoside, quercetin-3-O-rutinoside, and sophorin isolated from Toona sinensis Roem (Meliaceae), has clinically relevant functions as an anti-inflammatory and antioxidant agent [212]. Similar to quercetin, luteoloside, isolated from the medicinal plant *Gentiana macrophylla*, has oncosuppressive effects in humans [213].

#### 4.2.2. Magnolol

Magnolol, a plant that is widely used in traditional Japanese and Chinese medicines, is isolated from the root of magnolia officinalis. It is well known that magnolol has anti-inflammatory, anti-diabetic, anti-microbial, anti-neurodegenerative and anti-depressant properties. Recently, in vivo and in vitro studies have shown that the treatment of neuroblastoma cancer cells with magnolol can induce autophagy/mitophagy and apoptosis in treated cells. Importantly, blocking autophagy/mitophagy significantly enhances the anti-cancer effectiveness of magnolol, suggesting that targeting autophagy/mitophagy can be a promising strategy to overcome chemoresistance and to improve cancer therapy [214].

#### 4.2.3. Kaempferol

Kaempferol, a polyphenol flavonoid, is found in different fruits (e.g., grapes) and vegetables (e.g., tomatoes). Kaempferol modulates autophagy in noncancerous cells in order to protect cells against malfunction, and it induces cell death by enhancing autophagy via the elevation of the p-AMP-activated kinase protein, LC3-II, and Beclin1 in gastric cancer cells [111].

#### 4.2.4. Apigenin

Apigenin, a bioflavonoid, is widely present in fruits and vegetables, such as parsley, orange, tea, chamomile and seasonings. Apigenin has been shown to possess significant anti-inflammatory, antioxidant and oncosuppressive properties. In cancer cells, apigenin inhibits growth and proliferation through its preventive effects via the modulation of apoptosis and autophagy [112]. The mechanism underlying the anti-tumor effects of apigenin in hepatocellular carcinoma HepG2 cells is related to the induction of apoptosis and autophagy through the inhibition of the PI3K/AKT/mTOR pathway [113]. However, apigenin inhibits autophagy flux in the primary human epidermal keratinocytes (HEKs) and the cutaneous squamous cell carcinoma cell line COLO-16. Moreover, apigenin can enhance the effect of chemotherapeutic agents and reduce chemoresistance by inhibiting drug efflux [112]. Gao et al. [215] investigated the possibility of a chemo-sensitization effect of apigenin in a Dox-resistant HCC cell line (BEL-7402/ADM). Apigenin treatment enhances Dox sensitivity, induces microRNA-520b (miR-520b) expression and inhibits ATG7-dependent autophagy in these cells. ATG 7 acts as a potential target of miR-520b [215]. Moreover, combined with N-(4-hydroxyphenyl) retinamide, apigenin may suppress starvation-induced autophagy and promote apoptosis in human malignant neuroblastoma cells [216]. There is also evidence that suggests that apigenin can induce autophagic cell death in human papillary thyroid carcinoma cells [217]. Vitexin (apigenin-8-C-glucoside, c-glycosylated flavone) is found in various medicinal plants [218]. The biochemical properties of vitexin, such as its anticancer and antioxidant effects, are well-documented [112]. Vitexin has been reported to inhibit autophagy in a multi-drug-resistant (MDR) line of human colon cancer cells (HCT-116DR). Mechanistically, vitexin could reduce the level of autophagy in cancer cells (via the suppression of ATG 5 and Beclin1 expression levels) and simultaneously increase the apoptotic response through the enhancement of the cleavage of caspase-3 and -9 [219].

#### 4.2.5. Coffee and Tea: (−)-epigallocatechin-3-gallate (EGCG), Catechin and Epicatechin

Coffee and tea are the most consumed beverages worldwide, and have an impressive impact on the economies of the countries that produce them. Coffee is prepared from the seeds of coffee plants, genus Coffea, which include different species. Tea made from the leaves of the plant *Camellia sinensis* is a popular beverage [220]. An elegant study showed that caffeine, the main constituent of coffee beans and tea leaves, is a potent stimulator of hepatic autophagic flux; caffeine-induced autophagy involves the down-regulation of mTOR signaling and alterations in hepatic amino acids and sphingolipid levels. Caffeine also promotes AMPK-dependent autophagy through calcium-mediated pathways in skeletal muscle cells [221]. It can be found in quantities of up to 70–350 mg per cup of coffee and has been linked to numerous health benefits, such as a reduced risk of some forms of cancers, including breast cancer [222]. However, coffee consumption has been associated with a risk of the development of various forms of cancers, including CRC [223] and bladder cancer [224]. Via the inhibition of enzymatic activity of mTORC1, Pietrocola el al. [225] has shown that consumption of both natural and decaffeinated brands of coffee by mice increases autophagic flux (1-4 h after intake) in the liver, muscles and hearts of the treated animals. However, they concluded that caffeine is not only responsible for increased autophagy, but also polyphenols, such as chlorogenic acid (CGA), EGCG, (−)-epigallocatechin (EGC), (−)-epicatechin-3-gallate (ECG), catechin and (−)-epicatechin within coffee, and tea may have an even stronger effect on autophagy activation and the reduction in protein acetylation. CGAs can activate AMPK-dependent autophagy pathways. These products show the effect of ameliorating a variety of human diseases, such as cancers [114]. The cosmetic industry has shown a growing interest in these polyphenols, since they are able to extend longevity significantly under several stress conditions by reducing skin aging and age-related diseases [207]. EGCG upregulates AMPK activity in a dose-dependent manner, while the mTOR pathway is inhibited in hepatoma cells [115]. An increasing amount of evidence has shown that the dietary intake of proanthocyanidins plays an essential role in the chemoprevention or chemotherapy of tumors [226]. In vitro and in vivo toxicity experiments have demonstrated that proanthocyanidins have anticancer effects on various human cancers, such as CRC, pancreatic cancer, HCC, non-small cell lung cancer (NSCLC), squamous cell carcinoma (SCC), as well as head and neck squamous cancer. Grape seed proanthocyanidins, formed by the polymerization of catechins and/or epicatechins, induce autophagy by inducing the phosphorylation of the mitogen-activated protein kinase (MAPK) pathway and by reducing the expression of survivin, which is a member of the inhibitor of apoptosis (IAP) gene family in HepG2 cells [227].

#### 4.2.6. Genistein

Genistein, a natural isoflavone polyphenol, has been reported to exhibit multiple beneficial effects on human health, including anticancer properties that target multiple cancer cells, such as ovarian cancer and human breast MCF-7 cells [116] through several mechanisms, which include the induction of autophagic cell death [117]. Several studies found that genistein can potentiate the antitumor effects of chemotherapeutic agents (e.g., 5-FU, gemcitabine, cisplatin and oxaliplatin) by modulating the autophagic-apoptotic pathway. For instance, the combination of 5-FU and genistein can induce autophagic cell death in cancer cells by significantly altering the expression of two important molecules, Bcl-2 and Beclin1, which regulate autophagy [117]. The oncosuppressive effect of genistein is associated with the inhibition of PI3K-AKT signaling activation [118]. Ali et al. [228] also reported that genistein inhibits nuclear receptor co-repressor (N-CoR) misfolding, which is an important component in the activation of the oncogenic survival pathway in NSCLC, and was found to be associated with heat shock cognate 70 kDa protein (HSC70), which is a molecular chaperone in autophagy. Surprisingly, genistein induces the overexpression of TFEB, which is a master regulator of lysosomal biogenesis and an enhancer of autophagy protein expression [229]. Moreover, genistein-mediated suppression of mTOR increases dephosphorylation and the subsequent nuclear translocation of TFEB, which is associated with a significant increase in lysosomal content and activity in treated cancer and non-cancerous cells. Thus, genistein appears to be a potentially beneficial agent in the treatment of lysosomal storage diseases and cancers [230,231].

#### 4.2.7. Curcumin Derivatives

Curcumin has been used as a food colorant in dietary supplements and herbal medicines in Asian populations [119]. Curcumin has numerous pharmacological activities, including antioxidant and anticancer activities [112,120]. Curcumin has been reported to induce autophagy in chronic myeloid leukemia, malignant glioma, esophageal cancer, colon cancer, uterine leiomyosarcoma, ovarian cancer and lung adenocarcinoma via mechanisms related to the reduction in cell viability, proliferation, migration and invasion [119]. Curcumin also induces autophagy-associated apoptosis in mesothelioma and chronic myelogenous leukemia cells by modulating PI3K/AKT/mTOR and the nuclear factor kappa-light-chain-enhancer of activated B cells (NF-κB) signaling pathways [112]. Curcumin inhibits the growth of malignant gliomas, lung adenocarcinoma and melanoma cells in vitro and in vivo by downregulating the PI3K/AKT/mTOR signaling pathway and activating the AMPK pathway, and finally by promoting autophagy [112]. In addition, curcumin induces apoptosis in human malignant mesothelioma, which is an aggressive malignancy and is inherently chemo-resistant [112,121]. Curcumin-induced cell death is highly correlated with the enhancement of apoptosis or autophagy, mitochondrial membrane potential (MMP) and the activation of caspase-3. In addition, curcumin can reduce the expression of Bcl-2 proteins in K562 cells [122,123]. Curcumin has been shown to activate mitochondrial-mediated apoptosis and autophagy in adriamycin-induced human hepatoma G2 (HepG2) [123], due to the reduced proportion of Bcl-2/Bax protein and caspase-3 activation. Moreover, curcumin treatment can result in the mitochondrial fission of HepG2 cells, the reduction in MMP and autophagy activation [123]. It has been proposed that curcumin reverses cisplatin chemoresistance via the regulation of oxidative stress and autophagy flux in the MDR cell line A549/cDDP [112]. Curcumin also sensitizes MDR breast cancer cells to cisplatin treatnment and activates autophagy by suppressing the PI3K/AKT/mTOR pathway [112]. Interestingly, curcumin also regulates prosurvival autophagy in HCT116 cells that are mediated by the overexpression and nuclear translocation of TFEB and the inhibition of mTOR [121,232]. The monocarbonyl analog of curcumin, B19 or curcumin bis-dehydroxy, induces autophagy and apoptosis via the ER-stress route in ovarian and colon cancer cells [118]. The curcumin analogue, hydrazinobenzoylcurcumin, can also induce autophagic cell death in human non-small lung epithelial carcinoma (A549) cells [121]. Tetrahydrocurcumin, a major metabolite of curcumin, significantly reduces the activity of the PI3K/AKT/mTOR and MAPK signaling pathways and induces autophagic cell death in human leukemia HL-60 cells [233,234]. Another curcumin analogue, the 3,5-bis (2-hydroxybenzylidine) tetrahydro-4H-pyran-4-1 glutathione conjugate (EF25-[GSH]2), inhibits the growth of hepatocellular carcinoma in vitro and in vivo by modulating the autophagic pathway and enhancing apoptosis [235]. Besides activating autophagy, curcumin also exhibits time- or concentration-dependent inhibition of cell proliferation, autophagy and apoptosis in K562 cells, SKN and SK-UT-1 uterine leiomyosarcoma cells [236]. Curcumin therapy has been reported to mitigate autophagy and to reverse drug-resistance through the potent activation of Keap1 transcription, which is crucial for the erythroid 2 like 2 (Nrf2) signaling pathway [112,237].

#### 4.2.8. Resveratrol

Resveratrol has the potential to slow down the progression of many age-related diseases (ARDs), including different types of cancer. Resveratrol has potentially beneficial effects, including improving mitochondrial quality control and glucose tolerance through AMPK activation [118]. Several studies have suggested the growth inhibitory efficacy of resveratrol in several types of cancer cell, such as HCC, breast cancer, gastric cancer and leukemia [124]. Interestingly, resveratrol significantly inhibits breast cancer stem cell proliferation by inducing autophagy through the suppression of the Wnt/β-catenin signaling pathway. Resveratrol treatment in cancer cells results in autophagic cell death via multiple pathways, including JNK-mediated p62 expression, AMPK activation and the Beclin1-independent pathway [125]. Resveratrol has been reported to reduce AKT phosphorylation and mTOR signaling by p70S6K, which is a direct mTOR substrate. Resveratrol treatment decreases ER Ca^2+^ storage and store-operated calcium entry (SOCE), which induces ER stress, thereby activating AMPK and inhibiting the AKT/mTOR pathway [126]. Moreover, some studies have also suggested that resveratrol may potentially be useful in cancer chemotherapy for HCC and leukemia, when used in combination with other drugs, mainly due to its effect on apoptosis [126]. Rapamycin, in combination with resveratrol, significantly inhibits the growth of estrogen receptor-positive and estrogen receptor-negative breast cancer cells by preventing the activation of the AKT pathway, autophagy, and stimulating apoptosis [121]. In another study, resveratrol, in combination with the carfilzomib proteasome inhibitor (at low concentrations), synergistically increases apoptosis in myeloma cells through the simultaneous induction of autophagy [238]. This compound can also increase the susceptibility of melanoma, prostate and NSCLC cancers to chemotherapy [112]. In another study, resveratrol was reported to attenuate autophagy in cigarette smoke-induced cytotoxic stress responses in lung cells via the activation of SIRT1, which is a potent inducer of autophagy [121]. Pterostilbene (trans-3,5-dimethoxy-4-hydroxystilbene), a resveratrol analogue, triggers autophagy-induced apoptosis in cisplatin-resistant human oral cancer cells via the triggering of SIRT1 [239].

#### 4.2.9. Propolis Extract: Chrysin

Propolis is a complex resinous mixture produced by honeybees and has multiple pharmacological properties, including anticancer activity. Brazilian green propolis extract, which contains the active ingredients cinnamic acid derivative artepillin C, is an attractive agent for cancer treatments [127]. In addition, the ethanol extracts of Chinese and Brazilian green propolis have been reported to induce autophagy in prostate cancer CWR22Rv1 cells via the upregulation of LC3-II [127]. The apoptosis induced by artepillin C is exacerbated by cotreatment with autophagy inhibitors, such as CQ [127]. A number of studies have confirmed the biological properties of chrysin, including its anti-inflammatory and anti-tumor activity [240]. Chrysin is an effective component in sensitizing human glioblastoma cells (GBM8901) to temozolomide (TMZ). It inhibits TMZ-induced autophagy by reducing the expression levels of LC3-II, ATG7 and Beclin1, and by suppressing the expression of O6-methylguanine-methyltransferase (MGMT) DNA, which may be involved in chemoresistance to TMZ [112].

#### 4.2.10. Fisetin

Fisetin, a flavonoid polyphenol, is known to exhibit multiple pharmacological activities, including anti-inflammatory and anticancer activities in various cell types, such as prostate, colon, breast, and leiomyoma cells [128]. Fisetin induces autophagy and apoptosis in various cancer cells, such as pancreatic cancers and human melanoma, via ER stress-and mitochondrial stress-dependent pathways [128]. Treatment of prostate cancer cells with fisetin suppresses mTOR activity and downregulates the subunits Raptor, Rictor, PRAS40 and GβL, in addition to activating the mTOR repressor tuberous sclerosis complex 2 (TSC2). Fisetin has been shown to be a dual inhibitor of PI3K/AKT and mTOR in prostate cancer cells and in human NSCLC cells, as well as an inducer of autophagy in pancreatic cancer cells via ER stress- and mitochondrial stress-dependent pathways [129,130]. Fisetin has been reported to induce autophagic-programmed cell death rather than cytoprotective autophagy in human NSCLC, liver cancer, prostate cancer, laryngeal cancer, and uterine leiomyomas, all through apoptosis signaling pathways [129]. Fisetin’s effects on autophagy are cell-type-dependent, since this compound inhibits autophagy in HepG2 cells via the PI3K/AKT/mTOR and AMPK pathways [241].

#### 4.2.11. Rottlerin

Rottlerin, a traditional Indian subcontinent medicine, displays antioxidant properties and anticancer potential against different cancer cells, e.g., breast cancer, with various mechanisms, including the induction of autophagy and apoptosis [118]. Singh et al. [137] reported that 2 µM rottlerin (24 h treatment) activates autophagy in pancreatic cancer stem cells by inhibiting mTOR signaling. In prostate cancer stem cells, it represses mTOR, which is accompanied by an increase in the expression of ATG proteins, including ATG5, ATG7, ATG12 and Beclin1 [138,139]. In addition, it is a protein kinase C δ (PKC-δ)-selective inhibitor, which in turn leads to the suppression of NF-κB signaling and the consequent activation of autophagy in breast, pancreatic and colon cancer cells. [139]. Rottlerin inhibits NF-κB and activates AMPK in breast and colon cancerous cells, resulting in a significant reduction in cellular ATP levels and autophagy induction. Rottlerin-induced autophagy leads to apoptotic cell death by multiple signaling pathways, such as PKCδ/transglutaminase 2 (TG2)-dependent and -independent pathways in pancreatic cancer cells, PKCδ-independent mechanism in HT1080 human fibrosarcoma cells, and inhibition of PI3K/AKT/mTORC1 pathways in prostate cancer stem cells (CSCs) [138], breast CSCs [242] and human pancreatic CSCs [137].

In addition, a variety of polyphenolic natural compounds or nutraceuticals isolated from fruits, vegetables, spices, nuts, legumes, herbs, etc., also regulate autophagy signaling pathways and exhibit potent anticancer activities. For example, cucurbitacin B enhances the anticancer effects of clinical chemotherapeutic drugs, including cisplatin, gemcitabine, methotrexate, docetaxel, and gemcitabine. It induces autophagy and DNA damage, as evidenced by the increasing ROS formation and autophagic protein expression in MCF-7 breast cancer cells [131,132]. Wogonin exerts inhibitory growth effects on the SW48 CRC cells by inducing autophagic and apoptotic cell death via modulating the PI3K/AKT signaling pathways. Wogonin upregulates autophagic proteins such as LC3II and Beclin1, in addition to apoptotic proteins, such as caspase 3, 8 and 9 and Bax [125,133,134]. Morusin has been highlighted for its versatile potential against human pathologies, including cancer and immune dysfunctions. Morusin treatment leads to mTOR1 inhibition and the subsequent activation of AMPK, resulting in ULK1-mediated autophagy activation [135,136]. Naringin inhibits human gastric carcinoma, via the induction of autophagy, by activating Beclin1 and LC3-II via the activation of MAPKs pathways [243]. 6-C-(E-phenylethenyl) naringenin (6-CEPN) has been shown to suppress colon cancer cell proliferation via the induction of necrotic cell death and autophagy by the inhibition of c-Raf/MAPK (MEK)/ERK and PI3K/AKT/mTOR signaling pathways [244].

### 4.3. Terpenoids

Paclitaxel and its semisynthetic analogue docetaxel have been widely prescribed antineoplastic agents (approved by the FDA in 1992) over the past several decades for a broad range of malignancies, such as ovarian cancer, breast cancer, and NSCLC, either as a monotherapy or in combination with cisplatin. The anticancer activity of this drug is attributed to its unique mechanism of action, i.e., causing mitotic arrest in cancer cells, which leads to apoptosis through the inhibition of microtubule depolymerization [60]. However, resistance to paclitaxel has become a major limitation of clinical success [140]. While the molecule or key mechanism associated with paclitaxel resistance in cancers remains uncertain, paclitaxel’s regulation of autophagy is one reason. It has been reported that paclitaxel promotes autophagy in ovarian cancer, cervical cancer SiHa cells, lung cancer cells, gastric cancer BGC823 cells and bladder urothelial carcinoma (BUC) cells [60]. Paclitaxel treatment has also been found to induce autophagy in A549 cells, U87 glioma cells, human PC-3 prostate cancer and colon HT-29 cancer cells via the increasing expression levels of LC3-II, ATG5 and Beclin1 in a dose-dependent manner. Additionally, paclitaxel-induced autophagy has been reported to play a critical role in mediating caspase independent cell death. Paclitaxel also inhibits autophagy in breast cancer cells and cervical cancer cells [60,141], suggesting that paclitaxel has different effects on autophagy in various cancer cells. As paclitaxel may generate unacceptable levels of toxicity in normal cells in clinical settings, more experiments need to be focused on how to enhance its effectiveness as well as to reduce its toxicity.

#### 4.3.1. γ-Tocotrienol

The use of tocotrienols, such as α-tocotrienol, β-tocotrienol, γ-tocotrienol and δ-tocotrienol, as dietary supplements in Asian populations is considerably higher than in developed countries. Most obviously, tocotrienols, members of the vitamin E superfamily, are characterized by their antioxidant, anti-inflammatory and anticancer activity [121]. Tiwari et al. [142] found that γ-tocotrienol treatment in breast cancer cells could induce ER stress and concurrent autophagy-mediated cell death. Oridonin (7,20-epoxy-ent-kauranes), a diterpenoid isolated from the medicinal herb Rabdosia rubescens, has been shown to display potent anticancer activity against a wide ranges of cancer cell types, such as breast cancer cells, melanoma and cervical carcinoma cells by inducing autophagy-mediated apoptosis [142]. In combination with oridonin, γ-tocotrienol has been shown to synergistically induce autophagic and apoptotic effects in mouse breast cancer cells [142]. This combination significantly enhanced the expression of autophagy markers, including LC3B-II, Beclin1, ATG3, ATG7, ATG5-ATG12 and cathepsin D [142]. γ-tocotrienol treatment promotes apoptosis and autophagy in human prostate cancer PC-3 and LNCaP cells [245]. Tocomin, which is a mixture of naturally occurring tocotrienols (T3s), inhibits proliferation and induces apoptosis in breast cancer cells [246].

#### 4.3.2. Ursolic Acid

Ursolic acid exhibits antitumoral activity by inhibiting proliferation, suppressing DNA replication, inducing the release of Ca^2+^, and activating caspases in several cancers, including breast carcinoma, melanoma, leukemia, hepatoma and prostate cancer [121]. In vivo, ursolic acid inhibits the growth of HCT15 cells by modulating autophagy involving the JNK pathway [121]. The pro-autophagic effects of ursolic acid in the suppression of TC-1 cervical cancer cells and NSCLC cells have been reported to be mediated by LC3-II and ATG5, depending on the concentration. In addition, ursolic acid triggers autophagy in MCF7 breast cancer cells through ER stress [121,144]. In another study, ursolic acid induced autophagy and apoptosis in glioblastoma U87MG cells by three different mechanisms, including phosphorylated extracellular signal-regulated kinase (PERK)/eukaryotic initiation factor 2α (eIF2α)/C/EBP homologous protein (CHOP), calmodulin-dependent kinase protein kinase (CaMMK)/AMPK/mTOR, and inositol-requiring enzyme 1α (IRE1α)/JNK signaling [121]. Ursolic acid-induced autophagy in PC3 prostate cancer cells is mediated by the Beclin1 and AKT/mTOR pathways [121,144].

#### 4.3.3. β-Elemene

β-elemene inhibits the activity of the PI3K/AKT/mTOR/p70S6K1 pathway, thus triggering autophagy and apoptosis in human NSCLC A549 cells and human renal-cell carcinoma 786-0 cells [121]. In the treated cells, induction of autophagy is protective, since the inhibition of autophagy with CQ significantly enhances the antitumor effect of β-elemene [121]. β-elemene has been shown to have the potential to reverse chemotherapeutic drug resistance. For example, β-elemene increases the sensitivity of 5-fluorouracil in p53 wild-type CRC cells [145] and reverses the resistance to gefitinib in NSCLC [146].

#### 4.3.4. (−)-Guaiol

(−)-Guaiol is well known for its antibacterial activity [147]. (−)-Guaiol inhibits the proliferation of NSCLC cells by inducing autophagy via specifically targeting mTOR phosphorylation at serine 2481 signaling pathways [147].

#### 4.3.5. Sesquiterpene Lactones: F1012-2

Sesquiterpene lactones (SLs), such as F1012-2, thapsigargin, parthenolide, and isoalentolactone, are plant-derived constituents that have a variety of biological activities in inhibiting proliferation, migration, invasion and inducing apoptosis in different types of cancer cells, such as lung cancer, breast cancer, leukemia, and CRC [148]. F1012-2 isolated from a perennial herbaceous plant (Eupatorium lindleyanum DC) inhibits the cell growth of triple negative breast cancer (TNBC) (MDA-MB-231 and MDA-MB-468) [148]. The cell growth inhibitory mechanisms of F1012-2 in TNBC cells are demonstrated by inducing apoptosis in a caspase-dependent manner, as well as the activation of autophagy. Simultaneously, F1012-2-induced apoptosis is enhanced by the inhibition of autophagy [148]. Similarly, ergolide [247], anthecotulide [248], CLE-10 [249], elephantopinolide A-P [250], and bigelovin [251] activate apoptotic and autophagic pathways in malignant melanoma, breast cancer (MDA-MB-231), HCC and liver cancer cells, respectively. Calcium ions (Ca^2+^) are an essential factor for the regulation of autophagy, because it has been shown that Ca^2+^ release helps to drive membrane fusion to a particular area from the ER, in the vicinity of autophagosomes and lysosomes by binding with IP3R [42]. Thapsigargin (TG) causes the transient elevation of cytosolic Ca^2+^ release from ER stores and the depletion of intracellular Ca^2+^ stores in several cells types due to the potent and specific inhibition of intracellular Ca^2+^ ATPases (sarcoplasmic-/endoplasmic reticulum sarco/ER Ca^2+^ ATPase, SERCA) [252]. TG therapy also leads to necrotic cell death, which results from excessive damage to the mitochondrial pool and activation of autophagy independent mTOR pathways [252]. The role of TG in autophagy is debated, with earlier reports claiming both inductive and inhibiting effects [148]. TG inhibits autophagy by specifically blocking autophagosome–lysosome fusion, as well as autophagic flux by interfering with Rab GTPases and Rab7 function [148].

### 4.4. Saponin Compounds

#### 4.4.1. Tubeimoside-1

Tubeimoside-1 (TBMS1) has been proven to have potent anticancer activities in human prostate, lung, liver, cervical, and gastric cancer cells [149]. TBMS1 is identified as a potent activator of autophagy in human breast and liver cancer cells via LC3-II accumulation [150] and AMPK activation [151]. Inhibition of cytoprotective autophagy can enhance the cytocidal effect of TBMS1 in breast cancer cells by promoting apoptotic cell death [150]. TBMS1 inhibits cell proliferation in melanoma cells in vitro and tumorigenecity in vivo. Interestingly, TBMS1 inhibits cell proliferation by the activation of the MEK1/2-ERK1/2 pathway on the one hand, and it triggers cytoprotective autophagy in melanoma cells on the other hand. The strength of the two opposing forces determines the fate of the cells. TBMS1 also interacts with protein-tyrosine phosphatase 1B (PTP1B), which further hyperactivates MEK1/2-ERK1/2 cascades, leading to the inhibition of cell proliferation and the partial distortion of prosurvival autophagy [253]. Another interesting report suggests that TBMS1 exerts anticancer effects in lung cancer cells via blocking of the late-stage of autophagy flux via the impairment of lysosomal acidification through v-ATPase inhibition and the induction of apoptosis by lysosomal-dependent pathways. TBMS1 promotes mitochondrial fission and the dynamin-related protein (DRP1), which is a small GTPase-mediated fragmentation, and thereby leads to ROS accumulation. Impairment of lysosomal acidification blocks the removal of dysfunctional mitochondria and results in ROS accumulation; this causes further damage to the lysosomal membrane and leads to cathepsin B leakage from lysosomes. This leakage upregulates the Bax-mediated MOM potential (MOMP), and subsequently, cytosolic cytochrome c-mediated caspase-dependent apoptosis [254].

#### 4.4.2. Paris Polyphylla

The Paris polyphylla extract has been reported to inhibit cell growth, EMT and invasion in breast cancer, ovarian carcinoma and lung cancer cells [152]. Moreover, pennogenin 3-O-beta-chacotrioside and polyphyllin VI are active components of the ethanolic extract from *P. polyphylla* (EEPP), inducing cell death in DLD-1 human CRC via the upregulation of autophagy markers LC3-II and Beclin1. In addition, EEPP therapy, in combination with Dox, improves cytotoxicity in these malignant cells [152]. Diosgenin-enriched *P. polyphylla* rhizome extract (DPPE) shows cytotoxicity and anti-cancer activities in breast cancer cells [255].

#### 4.4.3. Ophiopogonin B

Ophiopogonin B has been verified to inhibit cell proliferation in numerous NSCLC cells. Ophiopogonin B induces autophagy in H157 and H460 cells and adenocarcinoma A549 by upregulating the conversion of LC3-I to LC3-II and increasing the expression of ATG3 and ATG5-ATG12 in treated cells [153].

#### 4.4.4. Betulinic Acid

Betulinic acid (BA) exhibits a variety of biological activities, including anticancer effects. The anticancer activity has been linked to its ability to directly trigger autophagy-mediated apoptosis via the mitochondrial pathway, such as MMP [154]. BA inhibits cell proliferation and induces apoptosis in CRC cells, HepG2 and SMMC-7721 HCC [155]. BA treatment induces autophagy via the inhibition of the AKT/mTOR signaling pathway. Blockage of autophagy enhances BA-induced proliferation inhibition and apoptosis in CRC cells [156]. BA inhibits breast cancer metastases by targeting glucose-regulated protein 78 (GRP78), a major chaperone in ER that is frequently strongly expressed in most solid tumors. This GRP78 chaperone contributes to the acquisition of metastatic phenotypes, including apoptosis resistance and drug resistance [256]. The reduced congener of BA, botulin, also has several pharmacologic effects, including anti-cancer effects. Betulin exhibits inhibitory effects on colorectal metastasis by inducing cell-cycle arrest and autophagy in metastatic CRC cells via AMPK and PI3K/AKT/mTOR signaling pathways. In addition, betulin induces caspase-dependent apoptosis via decreasing the phosphorylation of the MAPK signaling pathway in metastatic CRC cells [257].

### 4.5. Alkaloids

#### 4.5.1. Camptothecin

Camptothecin (CPT), a potent topoisomerase I inhibitor, displays oncosuppressive activities against leukemia, human colon cancer and a variety of solid tumor systems [157]. Interestingly, the combination of miR-15a and miR-16, which are two potent inducers of autophagy, enhances the chemotherapeutic efficacy of CPT against cancer cells [157]. CPT enhances c-Myc-mediated ER stress and ROS generation, leading to autophagy activation via the induction of Ca2-mediated AMPK and the JNK/activator protein 1 (AP-1) pathway [158]. CPT also generates ROS to modulate the AMPK/mTOR/ULK1 axis in order to finally promote protective autophagy in esophageal cancer [159]. The combination of CPT and pulsatilla saponin D, a powerful autophagy inhibitor, has a synergistic anti-breast cancer effect by interrupting autophagic-lysosomal function and promoting ubiquitous p62 mediated protein aggregation [258]. CPT derivatives, such as belotecan and methylenebis, also induce autophagy, but with different mechanisms. Belotecan induces autophagy by decreasing the level of p62, whereas methylenedes do not affect the level of p62, but upregulate the level of LC3-II. Interestingly, methylenebis and belotecan enhance the synergic antitumoral efficacy by inducing the apoptosis of tumor cells [259].

#### 4.5.2. Berberine

Berberine (BBR) has diverse pharmacological properties, including cancer modulation [160,161]. BBR induces autophagic cell death and apoptosis in human gastric cancer, glioblastoma multiforme (GBM), breast cancer, and hepatoma cells by the activation of Beclin1, as well as the inhibition of the mTOR signaling pathway [160,161]. BBR-associated photodynamic therapy induces autophagy and apoptosis in renal carcinoma cells [162]. In addition, BBR has been shown to induce autophagic cell death by increasing the binding capacity of GRP78 levels in cancer cells [160,161]. BBR sensitizes human HCC to ionizing radiation by blocking autophagy and cell-cycle arrest, which results in senescence [260].

#### 4.5.3. Tetrandrine

Tetrandrine (TET) possesses multiple pharmacological properties against a wide variety of cancers [163]. The antitumor effects of TET are associated with the induction of apoptosis and autophagy, the reversal of MDR, and the enhancement of radiation sensitization [163,164]. TET has been proven to be a potent broad-spectrum autophagy agonist with effects on a variety of cell lines, including triple-negative breast cancer cells, human HCC, nasopharyngeal carcinoma by inhibiting PI3K/AKT/mTOR signaling [164,165]. TET inhibits human PML-RARα-positive acute promyelocytic leukemia (APL) cell proliferation and induces autophagy by activating ROS generation and Notch1 signaling [261]. TET induces autophagy and apoptosis in a dose-dependent manner in pituitary adenoma (PA) cells. TET induces autophagy in these cells by down-regulating the MAPK/STAT3 signal at low concentrations (1.25 μM). However, at a higher dose (5.0 μM) of TET, pituitary adenoma (PA) cells partially die from caspase-dependent apoptosis [262]. Unlike an autophagy enhancer, Qiu et al. presented TET as a powerful lysosomal inhibitor, blocking the autophagic flux at the lysosomal degradation step in tumor cells [263]. TET increases the sensitivity of different cancer cells to gefitinib [264], cisplatin [265] and tamoxifen [266].

#### 4.5.4. Protopine

Protopine exhibits a number of pharmacological properties, including anticancer activities. Protopine suppresses the cell proliferation of colon cancer, inhibits cell adhesion in breast cancer and induces apoptotic cell death in prostate cancer [166]. Protopine is capable of activating p53-mediated transcriptional activity and promotes the stabilization of the p53 protein. Protopine induces autophagy by enhancing LC3-II turnover, along with reducing the levels of p62 in human colon cancer cells [166].

#### 4.5.5. Neferine

Neferine possesses antitumor activity via the suppression of cell proliferation and the inhibition of cell growth in different human cancers [167]. Neferine can induce autophagy in various cancer cells, such as human ovarian cancer via the inactivation of mTOR and the activation of p38 MAPK/JNK signaling pathways [168]. Neferine induces autophagic cell death in a panel of cancer cells, including apoptosis-defective and -resistant cancer cells or isogenic cancer cells, via Ca^2+^ mobilization through the activation of ryanodine receptor and ULK1/PERK and AMPK/mTOR signaling cascades [169]. Neferine also enhances the tumor necrosis factor (TNF)-related apoptosis-inducing ligand (TRAIL)-mediated autophagic cell death in human prostate cancer cells via the JNK pathway [267]. Furthermore, neferine provokes autophagy and apoptosis in human neuroblastoma cells by reducing the levels of focal adhesion kinase (FAK) and 70 kDa ribosomal S6 kinase 1 (S6K1) [268]. Neferine induces ROS-dependent mitochondrial mediated apoptosis by increasing the expression of proapoptotic proteins Bax, cytochrome c, cleaved caspase-3 and caspase-9. It also activates autophagy via increasing Beclin1, ATG4, ATG5 and ATG12, LC3-II expression levels in cervical cancer cells [269]. Additionally, neferine enhances cisplatin-induced autophagic cell death in human lung adenocarcinoma via downregulation of the PI3K/AKT/mTOR signaling pathway [270]. Further evidence suggests that the neferine anti-angiogenesis mechanism in high-grade serious ovarian carcinoma occurs by inducing autophagy through the inhibition of the mTOR/p70S6K pathway and the suppression of the polarization of M2 tumor-associated macrophages [271].

#### 4.5.6. Graveoline

Graveoline triggers autophagic cell death in skin melanomas via the elevation ROS generation [170].

### 4.6. Quinonoids

Quinonoid compounds participate in multiple biological oxidative systems by serving as prime links in electron transport chains of the metabolic pathways. This redox characteristic accounts for the inherent cytotoxicity of quinonoids.

#### 4.6.1. Thymoquinone

Thymoquinone (TQ) has been revealed to exert outstanding pharmacological potential, including anticancer effects in both in vitro and in vivo models. TQ exhibits a high anticancer efficacy against various cancers cells, such as breast, lung, colon, ovary, larynx cervical, and prostate cancer, as well as multiple myeloma, myeloblastic leukemia, glioblastoma and osteosarcoma [171]. While the impact of TQ has been studied in many types of cancer, the molecular mechanisms underlying its action are complex and paradoxical due to the multiple targets (e.g., carcinogen metabolizing enzymes, transcription factors, etc.) involved in tumorigenesis or development of drug resistance [172]. TQ inhibits the metastasis of renal cell cancer cells [173], breast cancer cells [174] and human renal carcinoma cells [272] by inducing autophagy via the generation of ROS and the activation of the AMPK/mTOR signaling pathway. By inducing the chemomodulatory potential, TQ synergizes gemcitabine anti-breast cancer activity against human breast adenocarcinoma and ductal carcinoma cells via modulating its apoptotic and autophagic activities [174]. A combination of TQ with cisplatin diminishes the resistance fraction of cisplatin and improves its anticancer activity against head and neck cancer cells [273]. Similarly, TQ, in combination with TMZ, which is currently part of the standard treatment for glioblastoma (GBM), potently inhibits the growth of human GBM cell lines by transcriptional impairment of autophagy and the activation of apoptosis [274]. TQ has been shown to induce caspase-independent autophagic cell death in colon cancer cells via mitochondrial dysfunction (by induction of MOMP and activation of JNK and p38) [121,275].

#### 4.6.2. Celastrol

Celastrol is known for its potent anticancer activities. It has been demonstrated that celastrol inhibits the proliferation of various cancer cells and the growth of tumors in preclinical mouse models [121]. Celastrol can induce paraptosis, autophagy and apoptosis in different cancer cells, including HCC and osteosarcoma cells by modulating multiple pathways such as ER stress [121,175,176,177]. Celastrol has been reported to induce autophagy and apoptosis in different tumor cells, such as glioma and gastric cancer via the ROS/JNK and AKT/mTOR signaling pathways [276,277]. Furthermore, co-treatment of NSCLC cells with celastrol and erastin, which are ferroptosis inducers, initiates ATG5/ATG7-dependent autophagy and PINK1/Parkin-dependent mitophagy via the generation of ROS, the disruption of MMP and the promotion of mitochondrial fission [278]. Celastrol also stimulates Ca^2+^-mediated autophagic cell death by inhibiting both SERCA and P-gp in MDR tumor cells [279]. In combination with afatinib, celastrol induces paraptosis and subsequent cell death in NSCLC via ER stress, ROS accumulation and mitochondrial Ca^2+^ overload [280]. Celastrol also induces lipophagy via the activation of the liver-X receptors α (LXRα)/ATP-binding cassette transporter A1 (ABCA1) pathway in clear cell renal carcinoma [281]. Celastrol promotes Nur77 translocation from the nucleus to the mitochondria, where it interacts with TNF receptor-associated factor 2 (TRAF2), a scaffold protein and E3 ubiquitin ligase that is important in inflammatory signaling [282]. In human prostate cancer cells, celastrol induces autophagy by targeting the androgen receptor (AR)/miR-101. Celastrol-induced autophagy inversely correlates with AR expression levels, which in turn suppresses miR-101 expression, and thereby augments prostate cancer cell death [283]. Celastrol acts as a sensitizing agent to TRAIL-initiated lung cancer cell death via ROS generation and a decrease in MMP [284].

#### 4.6.3. Pristimerin

Pristimerin has been reported to provide a variety of anticancer activities via triggering autophagy-mediated cell death through ROS production and the activation of the JNK signaling pathway [178,179].

#### 4.6.4. Plumbagin

Plumbagin has been shown to exert a wide spectrum of pharmacological effects, including anticarcinogenic action against a variety of human cancer cells [180]. Accumulating evidence shows that the anticancer effects of plumbagin are mainly attributed to the induction of autophagy and apoptosis through intracellular ROS generation, activation of the AMPK pathway and inhibition of the PI3K/AKT/mTOR pathway [181,182,183].

### 4.7. Omega-3 Polyunsaturated Fatty Acids (ω-3-PUFA)

The ω3-PUFAs mainly consist of eicosapentaenoic acid (EPA) and docosahexaenoic acid (DHA). The anticancer properties and mechanism of action of ω3-PUFAs have been demonstrated in several cancers; however, autophagic and apoptotic cell death are the main mechanisms of DHA-induced cytotoxicity in these tumor cells. DHA induces autophagy and apoptosis in human cancer cells harboring wild-type p53 [187] and in prostate cancer cells expressing mutant p53 [188] through p53/AMPK/mTOR signaling [189]. ω3-PUFAs, including high-dose DHA, cause apoptotic and autophagic cell death in GBM cell lines by upregulating the expression of p62 [285], inducing PARP cleavage and activating the AMPK/mTOR pathway [286]. Increasing evidence shows that ω3-PUFAs exhibit anti-melanoma activity. Treatment of pulmonary melanoma with DHA-rich algal oil induces autophagy by inactivating mTOR and activating the JNK pathway, resulting in significant suppression of cell outgrowth. [287]. DHA promotes immunogenic apoptosis by inhibiting the STAT3 pathway in human multiple myeloma cells with no toxicity in peripheral blood mononuclear cells (PBMCs) and dendritic cells (DCs). It also activates autophagy in PBMCs and DCs, which potentially act as immune boosts [288]. There is evidence that EPA and DHA also induce anticancer effects by means of their conversion to their corresponding ethanolamine derivatives in breast carcinomas. This is done by binding and activating different receptors and distinct signaling pathways [289]. For instance, DHA and EPA-dopamine conjugates, such as DHA-dopamine (DHADA) and EPA-dopamine (EPADA), inhibit cell growth and trigger autophagy and apoptosis in breast cancer cells via the peroxisome proliferator-activated receptor γ (PPARγ) [290]. Cotreatment of retinoic acid and ω-3 PUFA activates autophagy in breast cancer cells by activating the p38 MAPK signaling pathways [291]. DHA enhances the anticancer drug oxaliplatin-induced autophagic cell death via activating Sestrin 2 and increasing ER stress in CRC cells [292]. Co-treatment with DHA and vitamin E delta-tocotrienol (Delta-T3) reduces lipid droplet biogenesis and potentiates lipophagy in TNBC MDA-MB-231 cells, resulting in the mitigation of breast cancer malignancy [293].

### 4.8. Miscellaneous

#### 4.8.1. Trichostatin A

Several histone deacetylase inhibitors (HDACIs), such as trichostatin A (TSA), SAHA (also known as vorinostat) and depsipeptide, have been widely studied as cancer therapeutic agents. TSA-induced apoptosis is associated with multiple mechanisms, with the most likely being the modulation of autophagy. The molecular mechanisms underlying TSA-mediated autophagy are still not clear, and autophagic cell death remains controversial and most likely context-dependent. TSA is able to induce autophagy in human cancer cells through the inhibition of the mTOR pathway and enhancing forkhead box protein1 (FOXO1)-dependent pathways [184]. Histone deacetylase inhibitors, valproic acid and TSA induce apoptosis and autophagy in pancreatic cancer cells by increasing ROS production and triggering mitochondrial membrane depolarization, cytochrome c release and caspase 3 activation [185]. In cervical cancer cells, TSA (1 μM) induces autophagy by significantly suppressing protein arginine methyltransferase 5 (PRMT5) and transient receptor potential cation channel, subfamily V, member 6 (TRPV6) levels and enhancing stanniocalcin 1 (STC1) and JNK levels [186]. TSA suppresses cervical cancer cell proliferation and induces autophagic cell death through the regulation of the PRMT5/STC1/TRPV6/JNK axis. In NSCLC patients, TSA treatment enhances autophagy and reverses the chemoresistance of docetaxel or paclitaxel associated with insulin-like growth factor (IGF) binding protein-2 (IGF-BP2) expression, which has been shown to promote tumorigenesis, metastasis, and cancer stem cell expansion [294]. TSA reduces cell viability in cisplatin-resistant human ovarian cancer cells by inhibiting the volume-sensitive organic anion channel (VSOAC) via the induction of taurine transporter (TauT) activity and inducing autophagic cell death [295]. In combination with the autophagy inhibitor CQ, TSA synergistically exerts anti-tumor activity in H-ras transformed breast epithelial cells by blocking the mTOR-signaling pathway [296]. Co-treatment with the PI3K/mTOR dual inhibitor, BEZ235 and TSA significantly enhances autophagic cell death and induces anti-tumor activities in esophageal squamous cell carcinoma [297] and breast cancer [298] by depressing the PI3K/AKT/mTOR signaling pathway and upregulating the expression of LC3-II and Beclin1.

#### 4.8.2. 6-Shogaol

6-shogaol, an active constituent of dietary ginger, exerts anti-inflammatory and anticancer properties. Treatment with 6-shogaol inhibits autophagy flux by increasing p62 and LC3 II levels in liver cancer cells. However, 6-shogaol, in combination with TRAIL, induces apoptosis via triggering ROS, upregulating p53 expression and altering the mitochondrial transmembrane potential (MTP) of these cells [299]. The antitumor activity of 6-shogaol in HCC indicates that it enhances autophagy by activating ROS and ER stress-associated proteins, and induces apoptosis by activating caspase-3 in cancer cells [300]. 6-shogaol inhibits cell survival and stimulates autophagy by suppressing the AKT/mTOR pathway in human non-small cell lung cancer A549 cells [301]. 6-shogaol induces both autophagic and apoptotic cell death in colorectal adenocarcinoma HT-29 cells [302]. 6-shogaol shows anti-tumor effect in cervical carcinoma by triggering the mitochondrial pathway of apoptosis and downregulating the PI3K/AKT/mTOR pathway [303]. 6-shogaol treatment in breast cancer cells results in the suppression of proliferation by the significant induction of apoptosis and the inhibition of autophagy by the regulation of the Notch signaling pathway (Hes1 and CyclinD1 genes). Here, the inhibition of autophagy by 6-shogaol leads to the enhancement of breast cancer cell apoptosis [304]. However, 6-shogaol induces autophagic cell death in breast cancer cells and CSC-like spheroids via γ-secretase mediated down-regulation of Notch signaling (reduction in the expression levels of cleaved Notch1 and its target proteins Hes1 and Cyclin D1) [305]. In addition, 6-shogaol potentiates the anticancer efficacy of 5-FU, oxaliplatin, and irinotecan by activating apoptosis and autophagy in CRC cells in hypoxic/aglycemic (glucose starvation) conditions [306].

#### 4.8.3. 7-Trehalose

Trehalose is a disaccharide that is present in a wide variety of organisms, including bacteria, yeasts, fungi, insects, invertebrates and higher plants, where it may serve as a source of energy and carbon. Trehalose is a powerful autophagy inducer used for the clearance of misfolded proteins in cells and animal models of neurodegenerative diseases. Recently, it has been reported that trehalose induces cytoprotective autophagy and mitophagy (via enhancing autophagic flux) in prostatic cancer cells that show resistance to chemotherapy [307].

## 5. Concluding Remarks and Future Directions

Since the defects of autophagy have been linked to many human diseases, modulation of autophagy can prevent or treat various human cancers. It has been reported that autophagy plays a dual role in cancer, showing both anti-tumor and tumor promotion effects, depending on the carcinogenic stage, tissues involved, and microenvironment. Autophagy can prevent cancer through the clearance of oncogenic factors; however, it can promote cancer progression and metastasis in well-established tumors. The inhibition of autophagy in chemoresistant cancer cells can cause cell death, while triggering excessive and uncontrolled formation, and the accumulation of autophagosomes and auyolysosomes can stimulate apoptotic or cell death with autophagic features. The identification of natural products as modulators has already provided a significant understanding of the molecular mechanisms of autophagy. Recent advances in technology, such as high-throughput, image-based screens and high-powered scanning microscopy, have been developed by different labs to identify natural modulators, as well as dynamics of autophagy for quantitative analysis of autophagic machinery. As discussed throughout this review, the anticancer effects of the enormous range of natural products are related to their autophagy-modulating actions, either the suppression or induction of autophagy. However, several obstacles with experimental bases and clinical trials have hindered the direct execution of autophagy modulators in the clinic. It is likely that many of these obstacles can be circumvented upon the development of more selective autophagy modulators, more precise biomarkers of the autophagy machinery and more animal models of autophagy deficiency in vivo.

Practically, the take home action of autophagy in cancers depends on the type of tumor, stage of tumorigenesis, tumor niches, as well as the genetic, epigenetic and metabolic contexts. Discovery of the precise role of autophagy in tumor development and progression is vital for the development of novel anticancer therapies that target cancer eradication. In cancer therapy, numerous unresolved problems exist. On the one hand, conventional chemotherapy and radiation therapy leads to increased toxicity in normal cells and tumor cells, limiting their use for cancer treatment. Thus, the development new and effective therapeutic agents with minimal toxicity to normal cells is of paramount urgency. Natural products with anticancer action have expanded their consideration due to their favorable safety and efficacy profiles in clinical trials. On the other hand, tumor resistance is increasingly incorporated into chemotherapy, radiotherapy or targeted therapy. One of the reasons for this augmented resistance is the failure of apoptosis in cancer cells. Autophagy-mediated cell death may be an alternative solution for this problem. Natural products that have the capability to manipulate both autophagy and apoptosis may assist cancer cell death. In addition, natural products, in combination with conventional therapeutic tactics, may offer greater effectiveness against malignancy. Furthermore, natural products, such as magnolol [214], which manipulate both autophagy and mitophagy in cancer cells (dual mechanism) may be important therapeutic targets [308,309]. In addition, natural products that clear lipid droplets via lipophagy-dependent or -independent mechanisms may prevent cancer progression, as these organelles are formed in various cancers, including breast, prostate, and clear renal cell carcinoma, and play a role in cancer progression [310,311].

A recent article reported the successful application of computer-assisted methods in screening a unique and diverse collection of an inhouse library consisting of about 1000 individual natural products such as alkaloids, terpenoids, Diels–Alder-type adducts, isoflavones, chalcones, and cannabinoids. However, the compounds derived from these natural products have anticancer and antimicrobial properties, which could be controlled by autophagic machinery [312]. Interestingly, there is increasing evidence that autophagy proteins activate extracellular vesicles’ (EVs) biogenesis. The pathways between autophagy and EVs occur not only in mammalian cells, but also in plants. This can be significant in the context of cancer treatment through the interaction between natural products derived from EVs, which are loaded with oncosuppressive cargoes on mammalian cancer cells [313].

## Figures and Tables

**Table 1:** Examples of natural materials that modulate autophagy, including chemical structure.

Target Stage of Autophagy	Natural Product	Chemical Structure		Derivatives	Biological Sources	Autophagy-Related M/A	Traditional Indications	Affected Cancer Cell Type Tested	Remarks
**Autophagy inhibitors: Initiation and nucleation stage inhibitors**									
Nucleation	Wortmannin	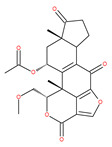		Fungal steroidal furan	Penicillium wortmannin, Talaromyces wortmannin KY12420	Inhibition of class III PI3K activity	N/A, toxic for prolonged uses	Experimental agent	Nonspecific inhibition both class I and III PI3K [46,47,48,49]
Nucleation	Sonolisib (PX-866)	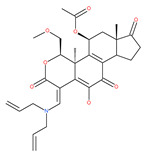		Furan-ring-opened	Wortmannin derivative	Inhibition of class III PI3K activity	-	NSCLC, SCCHN, Glioblastoma	Irreversible, and pan- PI3K inhibitor [50,51,52,53]
Initiation	Cycloheximide	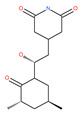		Bacterial actidione antibiotic	*Streptomyces griseus*	ULK1 suppression and activation of mTORC1.	Highly toxic	In vitro research only	Interference translocation step in protein synthesis [54,55]
Nucleation	Petrosaspongiolide M	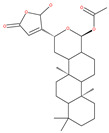		Marine γ-hydroxybutenolide terpenoid	Sponge, *Petrosaspongia nigra*	Down-regulation of Beclin1 levels	Preclinical studies	U937 cells	Inhibition both of proteasome and autophagy [56,57]
Nucleation	Harmine	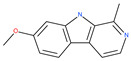		β-carboline alkaloid	*Peganum harmala* Lemon balm (*Melissa officinalis*)	Reduction in LC3-II expression	Anti-viral infection	A wide range of human cancers	Precise inhibitory mechanism of cellular enzymes remains elusive [58]
**Elongation and fusion stage inhibitors**									
Elongation and fusion	Vinblastine	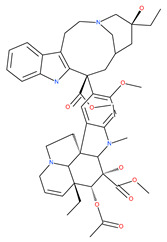		Vinca alkaloids	*Catharanthus roseus* (L.) or *Vinca rosea*	Depolymerization of whole MT network	Approved cancers	A wide range of human cancers	No protection the entry of autophagic flux [59,60]
Elongation and fusion	Vincristine	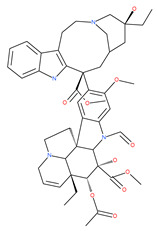		Vinca alkaloids	*Catharanthus roseus* (L.) or *Vinca rosea*	Depolymerization of whole MT network	Approved cancers	Leukaemia, lymphomas	No protection the entry of autophagic flux [59,60]
Elongation and fusion	Colchicine	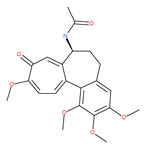		Colchicum alkaloid	Autumn crocus *Colchicum autumnale*	Depolarization and interruption of microtubule dynamics	Gout, Behçet’s disease, FMF, swelling	Lung cancer cells	Inducer of ROS mediated autophagy at clinically admissible concentration [59]
Elongation and fusion	Combretastatin	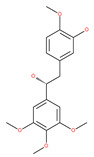		Cis-stilbenes (phenols)	South African tree *Combretum caffrum* (Combretacae)	Inhibition of MT polymerization	-	CT-26, Caco-2 and HT-29 cells	Vascular disrupting agents (VDA) [61,62,63,64]
Elongation	N-Acetyl-L-Cysteine	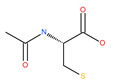		Prodrug of L-cysteine	Onion, *Allium cepa*	Limiting ROS and blocking ATG4	Chest pain, ALS, Alzheimer’s disease	A wide range of human cancers	In specific cases, autophagy activator [65,66]
Elongation	Xanthohumol	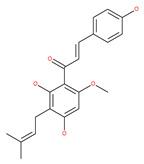		Natural Prenylated chalcone	Hop plant *Humulus lupulus* L.	Inhibition of VCP function; Up-regulation of p62 and LC3-II	Preclinical cancers	A wide range of human cancers	Activation of ER stress by suppressing of NF-κB, [67]
Elongation	Salvianolic acid B	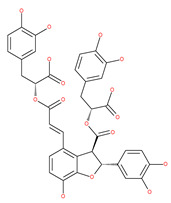		Stilbenoid	*Salvia miltiorrhiza* (Danshen)	Inhibition of LC3 lipidation	Cardiovascular-related disease, Fibrosis Disease	A wide range of human cancers	Induction of autophagy by suppressing the mTOR pathway [68,69,70,71,72,73]
Elongation	Deguelin	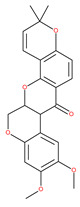		Natural rotenoid	African plant, *Mundulea sericea* (Willd)	Inhibition of LC3 lipidation	Preclinical cancer	A wide range of human cancers	Inhibition of autophagy flux by accumulating p62 [74,75]
**Docking and fusion stage inhibitors**									
Fusion	Monensin	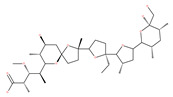		Polyether monocarboxylic acid antibiotic	*Streptomyces cinnamonensis*	Na^+^/H^+^ ionophore	Coccidiosis	A wide range of human cancers	Act as proton exchanger for K^+^/Na^+^ [76,77,78]
Fusion and degradation	Ionomycin	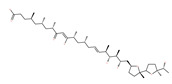		Natural ionophore	Bacterium *Streptomyces conglobatus*	Ca^2+^ ionophore, releases Ca^2+^ from intracellular stores	Antibiotic	Experimental agent	mTOR-independent autophagy inhibition [76,77,78]
Fusion	L-asparagine	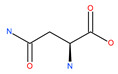		Nonessential amino acid	Dietary foods	Inhibition of efflux mechanism of lysosome	Development of brain	A wide range of human cancers	Low-asparagine diet can slow breast cancer metastasis
Fusion and degradation	Liensinine	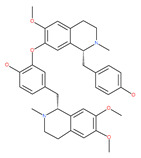		Isoquinoline alkaloid	Seed embryo of *Nelumbo nucifera* Gaertn	Impairment of RAB7A recruitment to lysosomes	Anti-arrhythmias, anti-hypertension, anti-pulmonary fibrosis	A wide range of human cancers	Unaffected lysosomal pH [79,80,81]
Fusion and degradation	Oblongifolin C	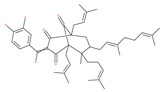		Polycyclic polyprenylated acylphloroglucinols	*Garcinia yunnanensis* Hu	Inhibition of lysosomal proteolytic activity	Antiseptics, antidepressants, and antibiotics	A wide range of human cancers	Guttiferone K is another active component of the plant causes autophagy induction [82,83,84,85,86]
**Autolysosomal acidification stage** **inhibitors**									
Fusion and degradation	Azithromycin	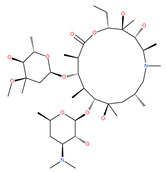		Macrolide antibiotic erythromycin analogue	*Actinomycete, Streptomyces erythreus (Saccharopolyspora erythraea)*	Prevention of autophagosomal acidification	Many pharmacological actions including COVID-19	A wide range of human cancers	Failure to kill intracellular mycobacteria in NTM, *Mycobacterium abscessus* in CF patients [87]
Fusion and degradation	Clarithromycin	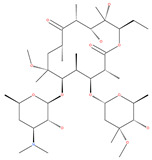		Macrolide erythromycin analogue	*Actinomycete, Streptomyces erythreus* (*Saccharopolyspora erythraea*)	Prevention of autophagosomal acidification	Antibiotic	A wide range of human cancers	Failure to kill intracellular mycobacteria [88]
Diffusion and degradation	Matrine	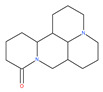		Quinolizidine alkaloid	Plants *Sophora flavescens Ait*	Inhibition of endosomal/lysosomal acidification	Anticancer, anti-inflammatory, antiviral actions	A wide range of human cancers	Induction of autophagy by activating the AMPK pathway [89,90,91]
Degradation	Elaiophylin	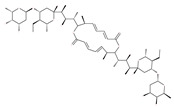		Macrodiolide antibiotic	*Streptomyces melanosporus*	Disruption of lysosomal degradation	Antibacterial and antihelminthic activities	A wide range of human cancers	Induction of LMP [92,93]
Degradation	Lucanthone	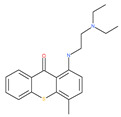		Thioxanthenones	Plant *Gentiana lutea* and mycelium of *Aspergillus stellatus* derivative	Blocking autophagic flux	Approved for schistosomiasis	A wide range of human cancers	A blocker of DNA base excision repair [94]
**Vacuolar H^+^-ATPase inhibitors**									
Fusion and degradation	Bafilomycin A1	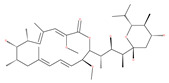		16-membered plecomacrolide	*Streptomyces griseus*	Lysosomal V-ATPase inhibition	Antibiotic, High toxicity profile	Experimental agent	Universal V-ATPase inhibitor (e.g., osteoclast) [95,96,97]
Fusion and degradation	Concanamycin A	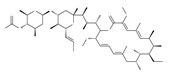		18-membered plecomacrolide	Mycelium *Streptomyces diastatochromogenes* S-45	Lysosomal V-ATPase inhibition	Antibiotic, Neoplasm	Experimental agent	Universal V-ATPase inhibitor (e.g., osteoclast) [98]
Fusion and degradation	Manzamine A	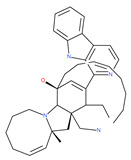		Manzamine alkaloid	marine sponges of the genera *Haliclona* sp., *Xestospongia* sp. and *Pellina* sp.	Lysosomal V-ATPase inhibition	Several pharmacological actions	A wide range of human cancers	v-ATPase inhibition is similar to bafilomycin A [99,100]
Fusion and degradation	Doxorubicin	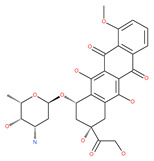		Anthracycline antibiotic	*Streptomyces peucetius, Streptomyces coeruleorubidus*	Lysosomal V-ATPase suppression	Antibiotic Antitumor	Approved for range of human cancers	Universal V-ATPase inhibitor [101,102]
Fusion and degradation	Cleistanthin-A	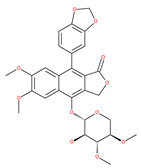		Diphyllin glycoside	Tropical plant *Cleistanthus collinus*	Lysosomal V-ATPase inhibition	Homicide	Experimental agent (In vitro)	Highly toxic and universal V-ATPase inhibitor [103]
Fusion and degradation	Archazolid A	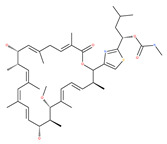		Macrocyclic polyketide	Myxobacterium Archangium gephyra, *Cystobacter violaceus*	Lysosomal V-ATPase inhibition	Antibiotic	Experimental agent (In vitro)	Potent and specific V-ATPase inhibitors [103]
**Lysosomal hydrolytic enzyme inhibitors**									
Degradation (partial)	Leupeptin	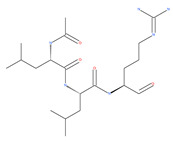		Tripeptide antibiotic	Multiple sources	Lysosomal thiolprotease and Ca^2+^-dependent calpain inhibitor	Serine protease inhibitor	Experimental agent (In vitro)	Agent for analyzing autophagy dynamics in vivo [104]
Degradation (partial)	Pepstatin A	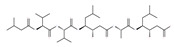		Hexapeptide metabolite	*Actinomycetes*	Lysosomal Aspartyl protease and cathepsin D inhibitor	Aspartyl protease inhibitor	Experimental agent (In vitro)	A reversible nonspecific inhibitor [105]
**Autophagy Inducers**									
Initiation	Rapamycin (sirolimus)	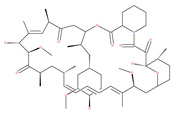		Macrocyclic lactone	Bacterium *Streptomyces hygroscopicus*	Specific inhibitor of mTOR	Many pharmacological actions	A wide range of human cancers	Suppression of mTORC2 function [106,107,108]
Initiation and nucleation	Quercetin	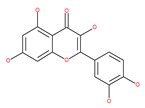		3, 3′, 4′, 5, 7-pentahydroxyflavone	Many foods, including fruits, vegetables and beverages	Induction of FOXO1 and ATG5 levels; AMPK activator	Dietary supplement; many pharmacological actions	A wide range of human cancers	Well-known antioxidant [109,110]
Initiation and nucleation	Kaempferol	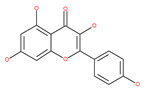		Tetrahydroxyflavone	Different in fruits and vegetables such as *Cuscuta chinensis* and *Hypericum perforatum*.	Induction of AMPK signaling	Dietary supplement; several pharmacological actions	A wide range of human cancers	Multipotential neuroprotective action in CNS diseases [111]
Initiation	Apigenin	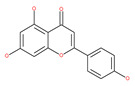		4′,5,7-trihydroxyflavone	Widely distributed in fruits and vegetables	Inhibition of the PI3K/AKT/mTOR pathway in HCC	Dietary supplement; several pharmacological actions	A wide range of human cancers	Inhibition of autophagy flux in in the HEKs and CSCC [112,113]
Initiation	EGCG, catechin and epicatechin	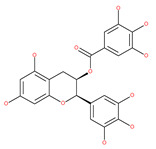 EGCG		Polyphenols	Coffee plant seeds, Tea leaves of *Camellia sinensis*	Activation of AMPK	Dietary supplement; several pharmacological actions	A wide range of human cancers	Amelioration of a variety of human diseases including cancers [114,115]
Initiation	Genistein	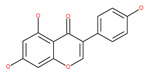		4′,5,7-trihydroxyisoflavone	Soybean and other legumes, such as *Vigna angularis*	Inhibition of the PI3K-AKT signaling pathway and N-CoR misfolding; induction of TFEB expression	Dietary supplement; several pharmacological actions	A wide range of human cancers	Beneficial agent in treatment of lysosomal storage diseases [116,117,118]
Initiation; elongation	Curcumin	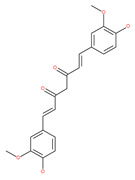		Turmeric polyphenol (beta-diketone)	Turmeric rhizome (*Curcuma longa L.*), *Curcuma zedoaria (Christm). Rosc.,* and *Curcuma petiolata*	Increases transcriptional activity of TFEB, which in turn suppresses mTOR and increases LC3 levels	Dietary supplement; many pharmacological actions	Clinical trial for cancers	In specific cases, autophagy inhibition [119,120,121,122,123]
Initiation	Resveratrol	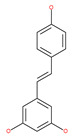		3,5,4′-trihydroxy-*trans*-stilbene (Stilbenoids)	Fruits such as berries, red grapes and peanuts, *Pterocarpus marsupium, Vitis amurensis* roots	Caloric restriction mimetic, AMPK activation	Dietary supplement; numerous pharmacological actions	A wide range of human cancers	Attenuation in cigarette smoke-induced autophagy (at higher concentrations) [118,124,125,126]
Nucleation	Chrysin	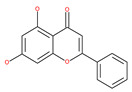		Dihydroxyflavone	Propolis, honey and plants such as *Passiflora* *caerulea, Passiflora incarnate*	Decrease in LC3-II, Beclin1 and ATG7 levels	Dietary supplement; numerous pharmacological actions	A wide range of human cancers	A potent inhibitor of aromatase [127]
Initiation	Fisetin	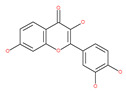		3,7,3’,4’-tetrahydroxyflavone	*Rhus verniciflua* Stokes, fruits and vegetables (apples, persimmons, grapes, kiwis, strawberries, onions	Activation of AMPK, suppression mTOR activity	Several pharmacological actions	A wide range of human cancers	Inhibition of autophagy in MCF7 breast cancer cells [128,129,130]
Initiation	Cucurbitacin B	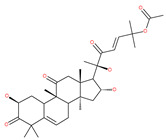		Tetracyclic triterpene	Cucurbitaceous plants	Induction of ROS formation	Numerous pharmacological actions	A wide range of human cancers	Protection against pressure-overload cardiac hypertrophy by inhibiting the AKT-mTOR pathway [131,132]
Initiation, nucleation	Wogonin	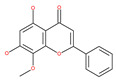		5,7-Dihydroxy-8-methoxyflavone	*Scutellaria baicalensis Georgi, Scutellaria radix*	*ER stress, upregulation of LC3-II and Beclin1*	Numerous pharmacological actions	A wide range of human cancers	Modulation of ER stress and autophagy and/or apoptosis in a cell-type- and context-dependent manner [133,134]
Initiation	Morusin	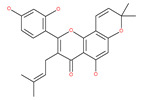		Mulberrochromene, prenylated flavonoid	Root bark of mulberry tree (*Morus alba* L., *Morus nigra* L, *M. australis)*	mTOR1 inhibition, AMPK activation	Antitumor, antioxidant and anti-bacteria property	A wide range of human cancers	Inhibition of NF-κB and STAT3 activity [135,136]
Initiation	Rottlerin (Mallotoxin or Kamala)	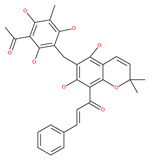		Polyphenolic ketone	*Mallotus philippinensis*	Inhibition of mTOR signaling and AMPK induction	Tapeworm, scabies and herpetic ringworm	A wide range of human cancers	Protein kinase C-δ inhibitor [137,138,139]
Nucleation	Paclitaxel	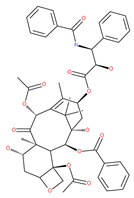		Polyoxygenated taxane class diterpenoid	Stem bark of the Pacific yew tree (*Taxus brevifolia* Nutt), bark of Pacific *Taxus chinensis*	Increase LC3-II, ATG5 and Beclin1 levels	FDA-approved for metastatic ovarian cancer	Lung-, ovarian- and breast cancers	Inhibition of autophagy in MCF-7 and SK-BR-3 breast cancer cells [60,140,141]
Initiation, nucleation	γ*-Tocotrienol*	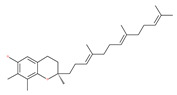		Unsaturated tail of tocotrienol	Abundant in rice bran oil, palm oil, and annatto seeds	AMPK activation, increasing LC3-II, ATG5 and Beclin1 levels	Many pharmacological actions including malabsorptive conditions	A wide range of human cancers	Vitamin E deficiency treatment [121,142]
Initiation, nucleation	Ursolic Acid	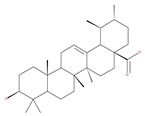		Pentacyclic triterpenoid	Plant, *Mirabilis jalapa*, apple peels	Increasing LC3-II, ATG5 and Beclin1 levels, inhibition of the mTOR pathway	Numerous pharmacological actions	A wide range of human cancers	mTOR-dependent and independent pathway followed [121,143,144]
Initiation	β-Elemene	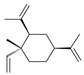		Sesquiterpene	*Rhizoma Curcumae, Curcuma wenyujin*	Inhibition of the mTOR pathway	-	KRAS mutant CRC cells	Rho kinase inhibitor [121,145,146]
Initiation	(–)-Guaiol	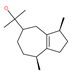		Sesquiterpene alcohol	*Guaiacum officinale Guaiacum sanctum*	Inhibition of the mTOR pathway	Antibacterial activity Gout	NSCLC cells	Blocking of mTORC2-AKT signaling [147]
Initiation	Thapsigargin	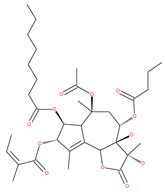		Sesquiterpene lactone	Root of umbelliferous plant *Thapsia garganica*	mTOR-independent Ca^2+^-dependent pathway	Many pharmacological actions	A wide range of human cancers	Inhibition of autophagosome–lysosome fusion [148]
Initiation nucleation	Tubeimoside-1	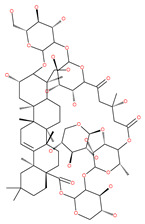		Triterpenoid saponin	*Bolbostemma paniculatum* (Maxim)	AMPK activation, inhibition of the mTOR pathway, increasing LC3-II level	Treatment of snake venoms and inflammation	A wide range of human cancers	[149,150,151]
Nucleation	Polyphyllin D	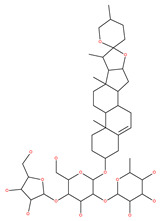		Steroidal saponin	*Paris polyphylla*	Increasing LC3-II and Beclin1 levels	Fevers, headaches, burns, and wounds, and treatment of snake venom	Human breast cancer cells	[152]
Nucleation	Ophiopogonin B	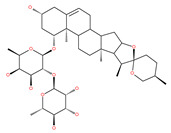		Saponin compound	*Radix Ophiopogon japonicus*	Increasing LC3-II and ATG5-ATG12 levels	Cardioprotective, diuretic and antibacterial activities	Gastric cancer, human cervical cancer	[153]
Initiation, nucleation	Betulinic acid	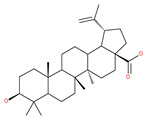		Lupane-type pentacyclic triterpenoid saponin	White birch bark, *Betula* *alba,* *Betula pubescens*	Inhibition of mTOR path 1way	Numerous pharmacological actions	A wide range of human cancers	Nutraceutical in anxiety and stress [154,155,156]
Initiation	Oleanolic acid	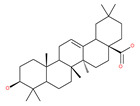		Pentacyclic triterpenoid	Olive oil, *Phytolacca americana, Syzygium* spp, garlic, etc.	ROS generation; AMPK activation and mTOR suppression	Numerous pharmacological actions	A wide range of human cancer cells	[143,144]
Initiation	Camptothecin	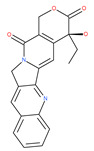		A quinoline type of alkaloid	Bark, stem and leaves of the Chinese happy tree, *Camptotheca acuminate, Chonemorpha fragrans*	Phosphorylation of AMPK	Anti-HIV activity	Lung, ovarian, breast, pancreas and stomach cancers	Potent topoisomerase I inhibitor [157,158,159]
Initiation, nucleation	Berberine	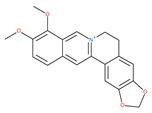		Isoquinoline alkaloid	*Coptis chinensis* Franch and *Rhizoma coptidis* herbs	Activation of Beclin1, inhibition of the mTOR pathway	Several pharmacological actions	A wide range of human cancers	An effective immunomodulator [160,161,162]
Initiation, nucleation	Tetrandrine	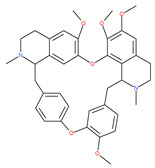		Bisbenzylisoquinoline alkaloid	Root of *Stephania tetrandra* S Moore	ROS production, elevation of Beclin1 and LC3-II levels	Many pharmacological actions	A wide range of human cancers	A potent lysosomal inhibitor [163,164,165]
Initiation nucleation	Protopine	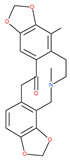		Benzylisoquinoline alkaloid	*Nandina domestica,* *Fumaria vaillantii*	p53 phosphorylation, Increase LC3-II level	Several pharmacological actions	Colon cancer cells	An activator of the p53 pathway [166]
Initiation	Neferine	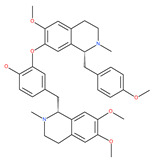		Bisbenzylisoquinoline alkaloid	Green seed embryos of *Nelumbo nucifera* Gaertn (lotus)	ROS generation, inactivation of th mTOR pathway	Several pharmacological actions	A wide range of human cancers	[167,168,169]
Initiation	Graveoline	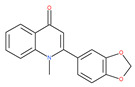		Alkaloid	*Ruta graveolens*	ROS generation, elevation of Beclin1 levels	Numerous pharmacological actions	Skin melanoma cells	[170]
Initiation	Peiminine	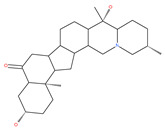		Alkaloid	*Fritillaria thunbergii*	Dephosphorylation of mTOR and AMPK activation	Numerous pharmacological actions	Colorectal cancer cells	Modulation of metabolic pathways
Initiation, Nucleation	Thymoquinone	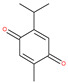		Edible monoterpene	Black cumin (seed) *Nigella sativa* L.	Induction of AMPK/mTOR signaling pathway, induction of LC3II expression	Dietary supplement; many pharmacological actions	A wide range of human cancers	Thymoquinone inhibits autophagy in glioblastoma cells [171,172,173,174]
Initiation, Nucleation	Celastrol (triperine)	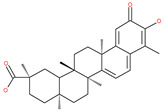		Quinone methide triterpenoid	*Tripterygium wilfordii* Hook (Thunder of God Vine), *Tripterygium regelii* (Regel’s threewingnut)	Induction of the ROS/JNK pathway, LC3B-II levels	Numerous pharmacological actions	A wide range of human cancers	Inhibition autophagy in prostate cancer cells [121,175,176,177]
Initiation	Pristimerin (Celastrol methyl ester)	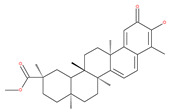		Quinone methide triterpenoid	*Celastraceae* and *Hippocrateaceae* families,	Induction of the ROS/JNK pathway	Numerous pharmacological actions	A wide range of human cancers	A potent and reversible monoacylglycerol lipase inhibitor [178,179]
Initiation	Plumbagin	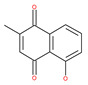		Naphthoquinone	Plants *Plumbago zeylandica* L (Chitrak), *P. europaea*, *P. rosea*, *Juglans regia*, *J. cinereal, J. nigra*	ROS generation, inhibition of the mTOR pathway	Numerous pharmacological actions	A wide range of human cancers	[180,181,182,183]
Nucleation	Emodin	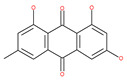		3-methyl-1,6,8-trihydroxyanthraquinone	Chinese herbs such as *Rheum palmatum* (rhubarb), *R. rhabarbarum L*	Induction of LC3II expression	Several pharmacological actions including antiviral activity against SARS-CoV-2	A wide range of human cancers	Attenuation of autophagy in in acute pancreatitis model
Nucleation	Gossypol	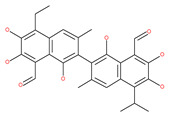		Phenolic natural product	Cotton seed (*Gossypium* spp.) *Malvaceae* family	BH3 mimetic	Male oral contraceptive, antimalarial property	A wide range of human cancers	-
Initiation	Anacardic acid	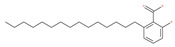		6-Pentadecyl-salicylic acid	Nutshell of the cashew tree, *Anacardium occidentale*	Stimulation of ER-stress, repression of AKT signaling	Gastric ulcer, gastritis and gastric cancer	Multiple tumor cells	-
Initiation, Nucleation	Withaferin-A	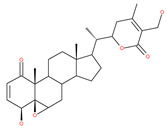		Steroidal lactone	Plant *Withania somnifera,* *Withania somnifera*(Indian Winter cherry)	ROS generation, induction of LC3-II	Numerous pharmacological actions including anti-inflammatory and antibacterial properties	A wide range of human cancers	Inhibition of lysosomal activity in breast cancer cells
Initiation	Magnolol	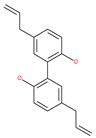		5,5’-diallyi-2,2’-dihydroxybiphenyl	Bark of *Magnolia officinalis* or *M. grandiflora*	Down-regulation of the Akt/mTOR pathway	Numerous pharmacological actions including anti-inflammatory effects	A wide range of human cancer cells	Mitochondria-targeted mito-magnolol is more effective than it.
Initiation, nucleation	α-Mangostin	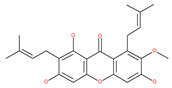		Xanthone derivative	*Garcinia mangostana Linn* (mangosteen fruit)	AMPK activation, induction of LC3-II	Numerous pharmacological actions including anti-aging	A wide range of human cancer cells	-
Initiation	Trichostatin A	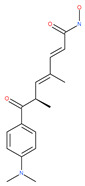		Natural Hydroxamate	*Streptomyces hygroscopius*	Inhibition of the mTOR pathway, enhancing the FOXO1-dependent pathway	Numerous pharmacological actions including antibiotic potential	A wide range of human cancer cells	Prolonged exposure (24h) leads to block autophagy [184,185,186]
Initiation	Antroquinonol	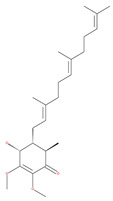		an enone	*Antrodia camphorata*	Inhibition of mTOR pathways	Numerous pharmacological actions	Various cancer cells	Inhibition of isoprenyl transferase activity
Initiation	ω-3 fatty acids (EPA, DHA)	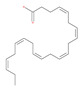 DHA	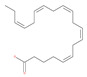 EPA	PUFAs	Dietary fats	ROS generation, inhibition of mTOR signaling	Many pharmacological actions	A wide range of human cancer cells	Undergoing clinical trials for ovarian cancer [187,188,189]
Transcription and elongation	All-trans retinoic acid	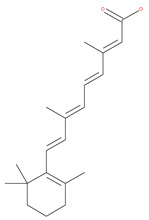		Vitamin A metabolite	Dietary fats	RARα activation; enhanced Beclin1 and LC3 II levels	Numerous pharmacological actions	Approved for cancers	In specific cases, autophagy inhibition [187,188,189]

**Table 2:** Ongoing and completed clinical trials with natural products for the treatment of cancer (the structures of these products are shown in Table 1).

Treatment Strategy		Disease/Conditions	Phase/Status	Identifier	Sponsor
Inhibitor	Other Intervention				
PX-866	Docetaxel	NSCLC; SCC	1/2/C	NCT01204099	Cascadian Therapeutics Inc.
	-	Advanced Solid Tumors	1/C	NCT00726583	Cascadian Therapeutics Inc.
	Cetuximab	Colorectal Carcinoma	1/2/C	NCT01252628	Cascadian Therapeutics Inc
	-	Glioblastoma	2/C	NCT01259869	NCIC Clinical Trials Group
Vinblastine	Sirolimus	CNS Tumors	1/C	NCT01135563	The Hospital for Sick Children
	CyP, Cape, Nivolumab	Childhood Lymphoma	1/2/R	NCT03585465	Centre Oscar Lambret
	Selumetinib Sulfate	Astrocytoma	3/R	NCT04576117	National Cancer Institute (NCI)
	Bevacizumab	Low Grade Glioma	2/R	NCT02840409	The Hospital for Sick Children
	Nivolumab	Hodgkin Lymphoma	2/R	NCT03580408	Lymphoma Academic Research Org.
	Dox, Bleomycin, Nivolumab	Hodgkin Lymphoma	1/2/R	NCT03033914	Memorial Sloan Kettering Cancer Center
	Dox, Bleomycin, Dacarbazine	Hodgkin Lymphoma	3/R	NCT03159897	Fondazione Italiana Linfomi ONLUS
	Cisplatin, TMZ	Skin Cancer	2/C	NCT00885534	Memorial Sloan Kettering Cancer Center
Vincristine	Cytarabine R-EphB4-HSA	Myelodysplastic neoplasm	1/R	NCT03519984	University of Southern California
	CyP Vinorelbine Actino D	Rhabdomyosarcoma	2/R	NCT04388839	H. Lee Moffitt Cancer Center
	Venetoclax	B ALL	1/2/R	NCT03504644	ECOG-ACRIN Cancer Research Group
	Inotuzumab Ozogamicin	Recurrent B ALL	1/2/R	NCT03851081	Roswell Park Cancer Institute
	TMZ	Ewing Sarcoma	2/R	NCT03359005	Peking University People’s Hospital
	Camrelizumab, Dacarbazine	Hodgkin Lymphoma	2/R	NCT04067037	Henan Cancer Hospital
	Carboplatin Irinotecan	Pilocytic Astrocytoma	1/2/R	NCT01837862	Julie Krystal
	Etoposide, Dox, CyP	Recurrent B ALL	1/R	NCT03991884	University of Washington
	Pegaspargase, CyP, etoposide	NK/T Cell Lymphoma	2/R	NCT04484506	Peking University
	Melphalan, etoposide	Retinoblastoma	2/3/R	NCT04681417	Institut Curie
	Brentuximab, Dox Rituximab	Hodgkin Lymphoma	2/R	NCT02398240	Mitchell Cairo
Colchicine	-	Hepatocellular Carcinoma	2/R	NCT04264260	Kaohsiung Medical University
	-	Hepatocellular Carcinoma	2/C	NCT01935700	Kaohsiung Medical University
CA-4P	Paclitaxel, Carboplatin	Cancer, Tumor	2/C	NCT00113438	Mateon Therapeutics
	Bevacizumab (Avastin)	Tumors	1/C	NCT00395434	Mateon Therapeutics
CA-2P	-	Neoplasm Metastasis	1/C	NCT00960557	Mateon Therapeutics
Fosbretabulin	-	Head and Neck Cancer	2/C	NCT00060242	Case Comprehensive Cancer Center
	Paclitaxel, Bevacizumab	Tumors	2/C	NCT00653939	Mateon Therapeutics
	-	Adult Solid Tumor	1/C	NCT00003698	University of Glasgow
	-	Adult Solid Tumor	1/C	NCT00003768	Case Comprehensive Cancer Center
	-	Neuroendocrine Tumors	2/C	NCT02132468	Mateon Therapeutics
	-	Neuroendocrine Tumors	2/C	NCT02279602	Mateon Therapeutics
NAC	-	Breast Cancer	1/C	NCT01878695	Thomas Jefferson University
	-	Ovarian Cancer	1/2/NR	NCT04520139	University of California, Irvine
	Simethicone	Stomach Neoplasms	4/C	NCT01653171	Pontificia Universidad Catolica de Chile
	-	Bronchial Carcinoma	4/C	NCT00196885	German Cancer Research Center
		Neurofibromatosis 1	2/R	NCT04481048	Children’s Hospital Med Center, Cincinnati
	Sodium thiosulfate	Esophageal Carcinoma	R	NCT04764643	Xijing Hospital of Digestive Diseases
Azithromycin	Hydroxychloroquine	Cancer and COVID 19	2/R	NCT04341207	Gustave Roussy, Cancer Campus, Grand Paris
	-	Adenomatous Polyposis	4/NR	NCT04454151	Tel-Aviv Sourasky Medical Center
	Cefixime	Cervical Papilloma	U	NCT02830230	Tata Memorial Hospital
Clarithromycin	Abemaciclib	Neoplasm Metastasis	1/C	NCT02117648	Eli Lilly and Company
	Prasterone	Multiple Myeloma	2/C	NCT00006219	Mayo Clinic
	Lenalidomide Dexamethasone	Multiple Myeloma	2/R	NCT04063189	The First Hospital of Jilin University
	Pomalidomide Dexamethasone	Multiple Myeloma	2/NR	NCT04843579	Weill Medical College of Cornell University
	Pomalidomide Dexamethasone	Multiple Myeloma	2/NR	NCT04302324	Weill Medical College of Cornell University
	Dexamethasone Lenalidomide	Plasma Cell Myeloma	2/C	NCT00445692	Fred Hutchinson Cancer Research Center
	Thalidomide dexamethasone	Multiple Myeloma	2/C	NCT00182663	Fred Hutchinson Cancer Research Center
	Dexamethasone Pomalidomide	Multiple Myeloma	2/C	NCT01159574	Weill Medical College of Cornell University
	Lenalidomide, dexamethasone	Multiple Myeloma	3/R	NCT04287660	First Affiliated Hospital of Soochow Uni
	Omeprazole, Amoxicillin,	Gastric MALT Lymphoma	NA/C	NCT00327132	National Health Research Institutes, Taiwan
	Amoxicillin, Metronidazole	B-cell Lymphoma	2/R	NCT02388581	National Health Research Institutes, Taiwan
	Ciprofloxacin, lansoprazole	CLL	2/C	NCT01279252	King’s College Hospital NHS Trust
	Amoxicillin, Metronidazole	Lymphoma	2/C	NCT00002682	M.D. Anderson Cancer Center
	Bismuth subcitrate Amoxicillin	Lymphoma	2/C	NCT00003151	EORTC
Lucanthone	TMZ, Radiation	Glioblastoma Multiforme	2/T	NCT01587144	Spectrum Pharmaceuticals, Inc
Dox	L-DOS47	Pancreas Cancer	1/2/R	NCT04203641	Helix BioPharma Corporation
	Irinotecan, 5-fluorouracil	Gastric Cancer	2/R	NCT04358341	Sixth Affiliated Hospital, Sun Yat-sen Uni
	Fludarabine	Ovarian Cancer	2/R	NCT03335241	Sun Yat-sen University
	CyP	Breast Cancer Patients	-	NCT04654195	Damanhour University
	PD-1	Bladder Cancer	2/R	NCT04101812	Tianjin Medical University Second Hospital
	-	Breast Cancer	2/R	NCT03933319	Chinese Academy of Medical Sciences
	Trastuzumab, paclitaxel	Breast Cancer	1/2/R	NCT03994107	Peking Union Medical College
	Bortezomib	Serous Carcinoma	2/R	NCT03509246	Seoul National University Hospital
	Bevacizumab, Pembrolizumab	Ovarian Cancer	1/R	NCT03596281	Gustave Roussy, Cancer Campus, Grand Paris
	CyP, Atezolizumab	Breast Cancer	2/R	NCT03164993	Oslo University Hospital
Rapamycin	-	Breast Cancer	2/R	NCT02642094	LuZhe Sun
	-	Bladder Cancer	2/R	NCT04375813	Emtora Biosciences
	-	Pancreatic Cancer	1/2/R	NCT03662412	Zhejiang University
	Nivolumab	Ewing Sarcoma	1/2/R	NCT03190174	Sarcoma Oncology Research Center, LLC
	-	Refractory Solid Tumors	4/R	NCT02688881	Samsung Medical Center
Quercetin	Fisetin, Dasatinib	Frailty, Childhood Cancer	2/NR	NCT04733534	St. Jude Children’s Research Hospital
	-	Squamous Cell Carcinoma	2/R	NCT03476330	Children’s Hospital Med Center, Cincinnati
	Green tea extract	Prostate Cancer	1/ANR	NCT01912820	Jonsson Comprehensive Cancer Center
EGCG	-	Colon Cancer	1/R	NCT02891538	University of Texas Health Science Center
	-	Breast Neoplasms	2/U	NCT02580279	Shandong Cancer Hospital and Institute
	-	Prostate Cancer	2/C	NCT00676780	Louisiana State University Health Sciences
Catechin	-	Adult solid tumor	1/C	NCT00091325	University of Arizona
EGCG	Clomiphene Citrate, Letrozole	Uterine Fibroids	1/R	NCT04177693	Yale University
Genistein	Sugar pill	Bladder Cancer	2/R	NCT01489813	Emory University
	Gemcitabine	Breast Cancer	2/C	NCT00244933	Barbara Ann Karmanos Cancer Institute
	Decitabine	NSCLC	1/2/C	NCT01628471	Uman Pharma
	-	Breast Cancer	2/C	NCT00290758	National Cancer Institute (NCI)
	Erlotinib HCl, gemcitabine HCl	Pancreatic Cancer	2/C	NCT00376948	Barbara Ann Karmanos Cancer Institute
Curcumin	-	Prostate Cancer	3/R	NCT03769766	University of Texas Southwestern Med Center
	-	Breast Cancer	1/R	NCT03980509	Medical University of South Carolina
	-	Breast Cancer	R	NCT03865992	City of Hope Medical Center
	-	Head and Neck Cancer	2/R	NCT04208334	Phramongkutklao College Hospital
	-	Prostate Cancer		NCT02064673	University of Texas Southwestern Med Center
	Pembrolizumab, Vit D, CyP	Endometrial Cancer		NCT03192059	University Hospital, Ghent
Resveratrol	Metformin	PCOS	2/NR	NCT04867252	Khyber Medical University Peshawar
	Simvastatin	PCOS	4/U	NCT02766803	Poznan University of Medical Sciences
	-	PCOS	U	NCT01720459	Poznan University of Medical Sciences
	Sirolimus	Lymphangioleiomyomatosis	2/U	NCT03253913	University of Cincinnati
Fisetin	Dasatinib, Quercetin	Childhood Cancer	2/NR	NCT04733534	St. Jude Children’s Research Hospital
β-elemene	EGFR-TKIs	NSCLC	2/U	NCT03123484	China Medical University
	-	Astrocytoma, Glioblastoma	3/R	NCT02629757	Sun Yat-sen University
Ursolic Acid	Curcumin	Prostate Cancer	1/NR	NCT04403568	University of Texas Health Science Center
Tocotrienol	Bevacizumab	Ovarian Cancer Recurrent	3/R	NCT04175470	Vejle Hospital
Paclitaxel	-	Breast Cancer	2/NR	NCT03959397	Jilin University
	Apatinib, Camrelizumab	Advanced Gastric Cancer	1/2/NR	NCT04286711	China Medical University
	Carboplatin	Prostate Cancer	1/2/NR	NCT04148885	Shanghai Jiao Tong University
	Pembrolizumab	Breast Cancer	2/NR	NCT03841747	Queen Mary University of London
	-	Breast Cancer	2/NR	NCT04194684	Chinese Academy of Medical Sciences
	Pyrotinib	Breast Cancer	2/NR	NCT04659499	Peking Union Medical College Hospital
	Oxaliplatin, Cape	Gastric Cancer	1/NR	NCT03977220	Shanghai Zhongshan Hospital
Mipsagargin	G-202, Tapsigargin pro-drug	Advanced Solid Tumors	1/C	NCT01056029	GenSpera, Inc.
Betulinic acid	-	Dysplastic Nevus Syndrome	1/2/S	NCT00346502	University of Illinois at Chicago
* CRLX101	Enzalutamide	Prostate Neoplasms	2/R	NCT03531827	National Cancer Institute (NCI)
* EP0057	Olaparib	Urothelial Carcinoma	1/2/R	NCT02769962	National Cancer Institute (NCI)
	Olaparib tablets	Ovarian Cancer	2/R	NCT04669002	Ellipses Pharma
Camptothecin	-	Adult Solid Tumor	1/C	NCT00059917	Memorial Sloan Kettering Cancer Center
	-	Solid Tumor	1/2/C	NCT00333502	NewLink Genetics Corporation
Berberine HCl	-	Colorectal Adenomas	2/3/R	NCT03333265	xiaohua li
	-	Colorectal Adenoma	2/3/C	NCT02226185	Shanghai Jiao Tong University
	Gefitinib	Lung Adenocarcinoma	2/U	NCT03486496	Fujian Cancer Hospital
	-	Colorectal Adenomas	2/3/U	NCT03281096	xiaohua li
Thymoquinone	-	Premalignant Lesion	2/C	NCT03208790	Cairo University
	Metformin	PCOS	2/3/C	NCT04852510	Saudi German Hospital—Madinah
R-(–)-gossypol	-	Adult Glioblastoma	2/C	NCT00540722	National Cancer Institute (NCI)
	-	Adrenocortical Carcinoma	2/C	NCT00848016	National Cancer Institute (NCI)
	TMZ	Brain and CNS Tumors	1/C	NCT00390403	Sidney Kimmel Com Cancer Center
	Paclitaxel, carboplatin	Follicular Lymphoma	1/C	NCT00891072	National Cancer Institute (NCI)
		Non-small Cell Lung Cancer	3/U	NCT01977209	Third Military Medical University
	Cisplatin, etoposide	Small Cell Lung Cancer	1/C	NCT00544596	National Cancer Institute (NCI)
Trichostatin A	-	Hematologic Malignancies	1/U	NCT03838926	Vanda Pharmaceuticals
Antroquinonol	-	Pancreatic Neoplasm	1/2/R	NCT03310632	Golden Biotechnology Corporation
	-	Non-small Cell Lung Cancer	1/C	NCT01134016	Golden Biotechnology Corporation
	-	Non-small Cell Lung Cancer	2/C	NCT02047344	Golden Biotechnology Corporation
	-	Acute Myeloid Leukemia	2/C	NCT03823352	Golden Biotechnology Corporation
EPA	AMR101	Colorectal Adenoma	1/2/R	NCT04216251	Massachusetts General Hospital
	AMR101 (VASCEPA)	Colon Cancer	2/R	NCT03661047	Mingyang Song
EPA-FFA	-	Adenomatous Polyposis	3/R	NCT03806426	S.L.A. Pharma AG
EPA	Tyrosine kinase inhibitor	Chronic Myeloid Leukemia	1/2/R	NCT04006847	Milton S. Hershey Medical Center
Icosapent Ethyl	-	Liver Metastasis	3/R	NCT03428477	Mark A Hull, PhD FRCP
DHA	-	Breast Cancer	2/R	NCT03831178	AHS Cancer Control Alberta
	EPA	NSCLC	NR	NCT04175769	AHS Cancer Control Alberta
	EPA	Lung Cancer	2/3/U	NCT01048970	National Institute of Cancerología
	Paclitaxel	Pancreatic Cancer	2/U	NCT00024375	Theradex
	Paclitaxel	Prostate Cancer	2/U	NCT00024414	Theradex
	Paclitaxel	Colorectal Cancer	2/U	NCT00024401	Theradex
ATRA	Lupron, 5-Azacitidine	Prostatic Neoplasms	2/R	NCT03572387	Icahn School of Medicine at Mount Sinai
	Anastrozole	Breast Neoplasm	2/R	NCT04113863	Mario Negri Inst for Pharm Research
	-	Leukemia	R	NCT01064557	Gruppo Italiano Malattie
	VEGFR inhibitor	Adenoid Cystic Carcinoma	2/R	NCT04433169	Shanghai Jiao Tong University
	Tranylcypromine, cytarabine	Acute Myeloid Leukemia	1/2/R	NCT02717884	Michael Luebbert
	-	APL	2/R	NCT01064570	Gruppo Italiano Malattie
	Arsenic Trioxide, Mylotarg	APL	2/R	NCT04793919	Associazione Italiana Ematologia
	Cytarabine, Arsenic Trioxide	AML	1/2/R	NCT03031249	Institute of Hematology and Blood Diseases

* CRLX101 is a nanoparticle-drug conjugate of CPT molecules; EP0057, a Nanoparticle CPT.

## Data Availability

Not applicable.

## Abbreviations

UPS	ubiquitin-proteasome system
ER	endoplasmic reticulum
ATG	Autophagy-related genes
FDA	Food and Drug Administration
IM	isolation membrane
ULK1	Unc-51-like autophagy activating kinase 1
PI3K	Phosphatidylinositide 3-kinase
mTORC1	mechanistic target of rapamycin complex 1
AMPK	AMP-activated protein kinase
MOM	Mitochondrial outer membrane
MOMP	MOM potential
ERES	ER exit sites
MAMs	mitochondria-associated ER membranes
PI3P	Phosphatidylinositol 3-phosphate
DFCP1	double FYVE domain–containing protein 1
Ubl	ubiquitin-like
LC3	MT-associated protein 1 light chain 3
LC3-PE	LC3-phosphatidylethanolamine
LIR	LC3 interacting regions
Ambra1	Activating Molecule In Beclin1-Regulated Autophagy Protein 1
p^62^/ SQSTM1	Sequestosome-1
MTs	Microtubules
ALR	autophagic lysosome regeneration
IP_3_R	inositol 1,4,5-trisphosphate (IP_3_) receptor
JNK	c-Jun N-terminal kinase
BH3	Bcl-2 homology (BH) domain 3
3-MA	3-methyladenine
MLCK	myosin light chain kinase
PLK1	Polo-like kinase 1
DNA-PK	DNA-dependent protein kinase
ATM	ataxia–telangiectasia mutated
NP	Nanoparticle
NCT	National Clinical Trial
PSM	Petrosaspongiolide M
EPI	Epirubicin
Bcl-2	B-cell lymphoma 2
TP	tea polyphenol
MTAs	MT-targeting agents
CA-4	Combretastatin A-4
CA-4P	CA-4 phosphate
NAC	N-acetyl cysteine
ROS	reactive oxygen species
rhArg	recombinant human arginase
5-FU	5-fluorouracil
*VCP*	Valosin Containing Protein or p97
XN	xanthohumol
Sal A	salvianolic acid A
Sal B	salvianolic acid B
SCC	squamous cell carcinoma
CCC	circulating cancer cells
PTEN	phosphatase and tensin homolog deleted on chromosome 10
EMT	epithelial-mesenchymal transition
AKT	protein kinase B (PKB)
HIF-1α	hypoxia-inducible factor 1α
VEGF	Vascular endothelial growth factor
HCC	hepatocellular carcinoma
CRC	colorectal cancer
Dox	Doxorubicin
Cdk4	cyclin-dependent kinase 4
Hsp-90	heat shock protein-90
PARP-1	poly (ADP-ribose) polymerase 1
ALL	acute lymphoblastic leukaemia
CQ	Chloroquine
Baf A1	Bafilomycin A1
OC	Oblongifolin C
GUTK	guttiferone K
v-ATPase	vacuolar-type ATPase
LMP	lysosomal membrane permeabilization
SAR	structure-activity relationship
CR	Caloric restriction
SIRT1	Sirtuin 1
BNIP3/BNIP3L	BCL2/adenovirus E1B 19 kDa protein-interacting protein 3/ligand
STAT3	Signal transducer and activator of transcription 3
RAGE	receptor for advanced glycation end products
MDR1	multidrug resistance protein 1
P-gp	P-glycoprotein
NLRP3	NLR family pyrin domain containing 3
EGCG	(−)-epigallocatechin-3-gallate
CGA	chlorogenic acid
EGC	(−)-epigallocatechin
ECG	(−)-epicatechin-3-gallate
NSCLC	non-small cell lung cancer
MAPK	mitogen-activated protein kinase
N-CoR	nuclear receptor co-repressor
HSC70	Heat shock cognate 71 kDa protein
TFEB	transcription factor EB
NF-κB	nuclear factor kappa-light-chain-enhancer of activated B cells
MMP	mitochondrial membrane potential
Nrf2	Nuclear Factor, Erythroid 2 Like 2
ARDs	age-related diseases
CRM	caloric restriction mimetic
p70S6K	phosphorylation of p70 ribosomal protein S6 kinase
SOCE	store operated calcium entry
TMZ	Temozolomide
MGMT	O6-methylguanine-DNA methyltransferase
TSC2	*Tuberous Sclerosis Complex 2*
PKC-δ	protein kinase C δ
TG2	Transglutaminase
CSCs	cancer stem cells
6-CEPN	6-C-(E-phenylethenyl)naringenin
BUC	bladder urothelial carcinoma
PERK	phosphorylated extracellular signal-regulated kinase
eIF2α	Eukaryotic initiation factor 2α
CHOP	C/EBP homologous protein
CaMMK	Calmodulin- dependent kinase protein kinase
IRE1α	inositol-requiring enzyme 1α
TNBC	triple-negative breast cancer
MPT	mitochondrial permeability transition
SERCA	Sarcoplasmic-/ endoplasmic reticulum sarco/ER Ca^2+^ ATPase
TG	Thapsigargin
TBMS1	Tubeimoside-1
ERK1/2	Extracellular signal-regulated kinase 1/2
PTP1B	protein-tyrosine phosphatase 1B
DRP1	dynamin-related protein
EEPP	ethanolic extract from *P. polyphylla*
DPPE	Diosgenin enriched *P. polyphylla* rhizome extract
BA	Betulinic acid
GRP78	glucose-regulated protein 78
CPT	Camptothecin
AP-1	Activator protein 1
miR-15a	microRNA-15a
BBR	Berberine
PDT	photodynamic therapy
TET	Tetrandrine
APL	acute promyelocytic leukemia
PA	pituitary adenoma
TRAIL	Tumor necrosis factor (TNF)-related apoptosis-inducing ligand
TRAF2	TNF receptor-associated factor 2
FAK	focal adhesion kinase
S6K1	70-kDa ribosomal S6 kinase 1
TQ	Thymoquinone
GBM	glioblastoma multiforme
LXRα	liver-X receptors α
ABCA1	ATP-binding cassette transporter A1
ccRCC	clear cell renal cell carcinoma
AR	androgen receptor
ω3-PUFAs	omega-3 polyunsaturated fatty acids
EPA	eicosapentaenoic acid
EPADA	EPA-dopamine
DHA	docosahexaenoic acid
DHADA	DHA-dopamine
MM	multiple myeloma
PBMCs	peripheral blood mononuclear cells
DCs	dendritic cells
PPARγ	peroxisome proliferator-activated receptor γ
Delta-T3	vitamin E Delta-tocotrienol
HDACIs	histone deacetylase inhibitors
TSA	trichostatin A
SAHA	suberoylanilide hydroxamic acid
FOXO1	forkhead box protein1
PRMT5	protein arginine methyltransferase 5
TRPV6	transient receptor potential cation channel, subfamily V, member 6
STC1	stanniocalcin 1
IGFBP2	insulin-like growth factor (IGF) binding protein-2
VSOAC	volume-sensitive organic anion channel
TauT	taurine transporter
MTP	mitochondrial transmembrane potential
CLL	Chronic Lymphocytic Leukaemia
ANT	Active, not recruiting
T	terminated
C	Completed
S	Suspended
R	Recruitment
NR	Not yet recruiting
U	Unknown
ATRA	all-trans retinoic acid
CyP	Cyclophosphamide
Cape	Capecitabine
PCOS	Polycystic ovarian syndrome
FME	familial Mediterranean fever
HEKs	human epidermal keratinocytes
CSCC	cutaneous squamous cell carcinoma cell
NTM	nontuberculous mycobacteria
LYNUS	lysosomal nutrient sensing machinery
